# Large scale phenotype imputation and in vivo functional validation implicate *ADAMTS14* as an adiposity gene

**DOI:** 10.1038/s41467-022-35563-0

**Published:** 2023-01-19

**Authors:** Katherine A. Kentistou, Jian’an Luan, Laura B. L. Wittemans, Catherine Hambly, Lucija Klaric, Zoltán Kutalik, John R. Speakman, Nicholas J. Wareham, Timothy J. Kendall, Claudia Langenberg, James F. Wilson, Peter K. Joshi, Nicholas M. Morton

**Affiliations:** 1 Centre for Cardiovascular Science, Queen’s Medical Research Institute, University of Edinburgh, Edinburgh, EH16 4TJ UK; 2 Centre for Global Health Research, Usher Institute, University of Edinburgh, Edinburgh, EH8 9AG UK; 3 MRC Epidemiology Unit, Institute of Metabolic Science, University of Cambridge, Cambridge, CB2 0QQ UK; 4 Institute of Biological and Environmental Sciences, University of Aberdeen, Aberdeen, AB24 2TZ UK; 5 MRC Human Genetics Unit, Institute of Genetics and Cancer, University of Edinburgh, Edinburgh, EH4 2XU UK; 6 Centre for Primary Care and Public Health, University of Lausanne, Lausanne, 1010 Switzerland; 7 Swiss Institute of Bioinformatics, Lausanne, 1015 Switzerland; 8 Centre for Energy Metabolism and Reproduction, Shenzhen Institutes of Advanced Technology, Chinese Academy of Sciences, Shenzhen, China; 9 Shenzhen Key Laboratory of Metabolic Health, CAS Centre of Excellence in Animal Evolution and Genetics, Kunming, China; 10 Centre for Inflammation Research, University of Edinburgh, Edinburgh, EH16 4TJ UK; 11 Computational Medicine, Berlin Institute of Health (BIH) Charité University Medicine, Berlin, Germany

**Keywords:** Genome-wide association studies, Genetic variation

## Abstract

Obesity remains an unmet global health burden. Detrimental anatomical distribution of body fat is a major driver of obesity-mediated mortality risk and is demonstrably heritable. However, our understanding of the full genetic contribution to human adiposity is incomplete, as few studies measure adiposity directly. To address this, we impute whole-body imaging adiposity phenotypes in UK Biobank from the 4,366 directly measured participants onto the rest of the cohort, greatly increasing our discovery power. Using these imputed phenotypes in 392,535 participants yielded hundreds of genome-wide significant associations, six of which replicate in independent cohorts. The leading causal gene candidate, *ADAMTS14*, is further investigated in a mouse knockout model. Concordant with the human association data, the *Adamts14*^−/−^ mice exhibit reduced adiposity and weight-gain under obesogenic conditions, alongside an improved metabolic rate and health. Thus, we show that phenotypic imputation at scale offers deeper biological insights into the genetics of human adiposity that could lead to therapeutic targets.

## Abstract (web summary)

Our understanding of the genetic contribution to human adiposity is incomplete, as few studies measure adiposity directly. Here, the authors impute whole-body imaging adiposity phenotypes in large biobanks, enhancing their power to discover genes driving human adiposity, and further investigate one such gene using a mouse model.

## Introduction

Obesity is the fifth leading cause of death and affects more than 600 million adults worldwide^1^. Although it is simply defined as a body mass index (BMI) over 30 kg/m^2^, the presentation of the condition is heterogeneous. Adipose tissue can be distributed in many different ways throughout the human body and is often categorised into two main patterns; central and lower-body adiposity. The former is characterised by excess visceral adipose tissue (VAT) surrounding the intra-abdominal organs and greatly increases the chances of developing disease^2^. Conversely, lower-body adiposity and particularly gluteofemoral subcutaneous adipose tissue (SAT), reduces disease risk^3,4^. Anthropometric measures, such as BMI and waist-hip ratio (WHR), offer simple and non-invasive ways to infer adiposity. They are easy to attain and calculate but are crude representations of the underlying body shape. Bioelectrical impedance analysis (BIA) is another widely used low-cost method for inferring whole-body fat content with some accuracy^5^. Dual-emission X-ray absorptiometry (DXA) scans are a rapid and precise whole-body imaging technology. Unlike other imaging technologies, DXA scans offer the possibility of whole-body, as well as regional adiposity analyses^6^ and it has been shown that both whole-body and regional DXA adiposity measures associate with an increased mortality risk^7^. In addition, DXA measures compared to the above anthropometric measures, correlate more strongly with cardiometabolic risk factors^8^.

Even though facilitated by today’s obesogenic environments, genetic predisposition also plays an important role in the development of obesity^9^. Genome-wide association studies (GWAS) have been very successful at identifying genomic loci that associate with measures of obesity. Since the first large GWAS meta-analysis^10^, which led to the identification of the first and strongest BMI-associated locus to date, *FTO*^11^, the number of SNP associations with obesity has now reached over a thousand^12,13^. However, very few functional genomics and experimental studies have been conducted on these GWAS loci, which forms a bottleneck in elucidating the role of these association signals in human health and disease. At the same time, obesity GWAS have primarily focused on easily accessible anthropometric phenotypes, such as BMI and WHR, and not on disease-relevant adiposity endophenotypes, due to the associated expense. This has led to more variants of minute effect being discovered, which amount to incremental increases in the proportion of the accountable trait variance^13,14^. Therefore, focusing on phenotypes that assay body composition more directly could help explain more of the observed trait variation.

## Results

### GWAS of imputed DXA phenotypes

**Fig. 1:**
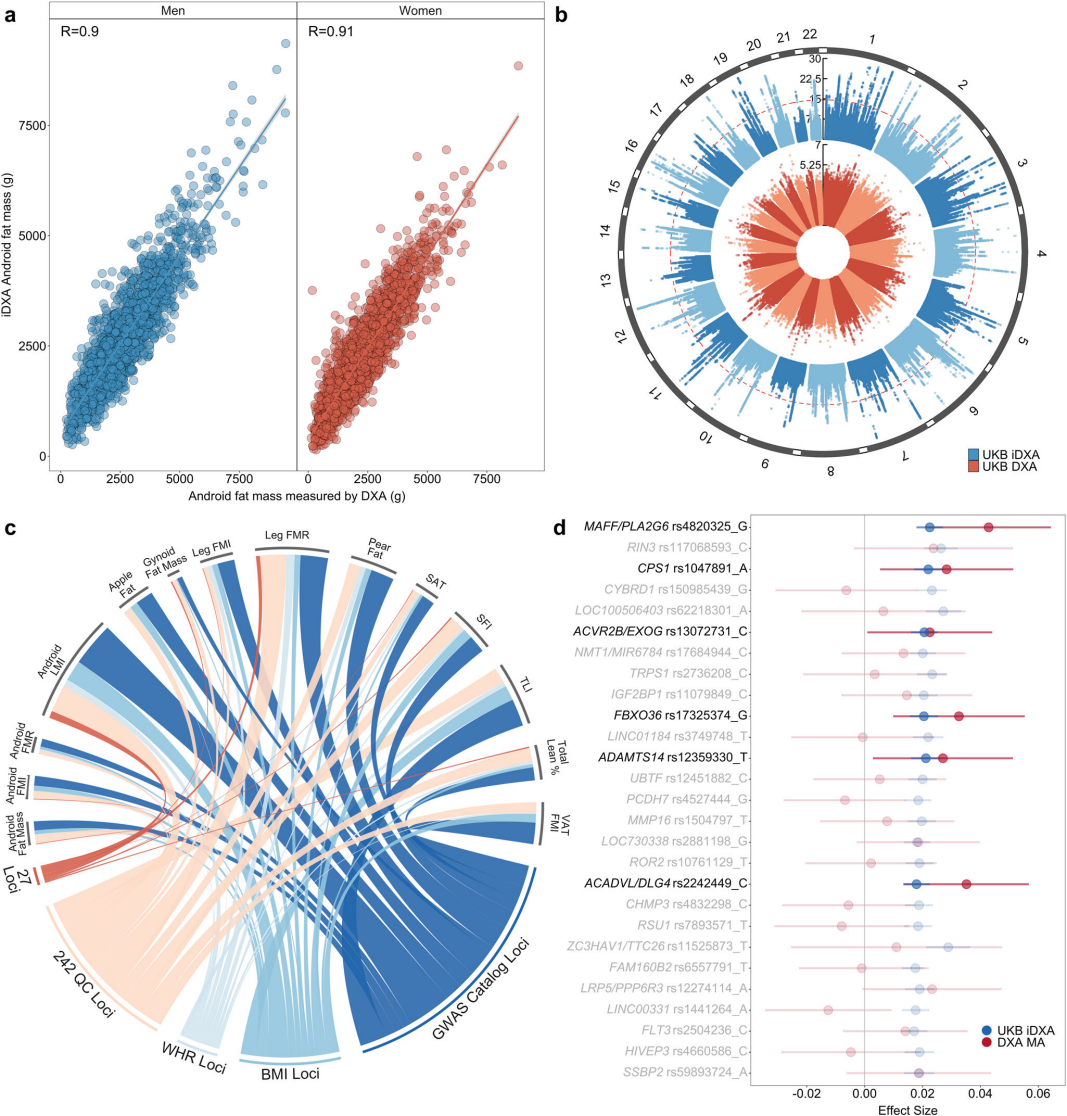
iDXA phenotypes provide accurate representations of DXA and much greater GWAS power leading to the identification of replicable genomic loci. **a** Example iDXA sex-separated prediction models as a function of DXA-measured Android fat mass. Correlations between DXA and iDXA given as Pearson’s R. Predictions for all other iDXA phenotypes can be found in Supplementary Data 1. **b** GWAS loci associated with collated DXA and iDXA phenotypes in the UKB cohort. DXA GWAS were conducted on the 4366 participants that were used in the prediction models, while iDXA GWAS were conducted in the rest of the cohort, excluding these 4366 individuals. iDXA *P* values capped at 10^−30^ for visibility. Red line indicates the multiple-testing adjusted GWS threshold, set at 1.25 × 10^−8^. Manhattan and QQ plots for each GWAS can be found in Supplementary Fig. 1. **c** Filtering and exclusion of identified loci based mainly on known associations with other phenotypes. Cumulative number of overlapping GWS SNPs (4764) across all iDXA GWAS were screened against known obesity associations in the GWAS Catalogue and for discoverable associations with BMI or WHR, within UKB. The remaining SNPs mapped to 242 QC novel loci, 27 of which were replicable for a replication sample size of 18,000 DXA-measured participants (Supplementary Data 3). **d** SNP effect sizes for the 27 replicable SNPs in Discovery (iDXA) and Replication (DXA meta-analysis (MA)). Genes annotated by proximity (±500 kb) to the lead-associated variant. Effect sizes in Discovery (iDXA) given as positive values for the corresponding allele and shown as beta ± 95% confidence intervals. The six highlighted SNPs were replicated successfully in the measured-DXA MA cohort (FDR < 10%). Detailed locus and phenotype information is given in Table 1, and replication summary statistics are given in Supplementary Data 4.

We used proxies of body composition within the UK Biobank (UKB) cohort to try and estimate some of the underlying DXA adiposity phenotypes. The DXA measures were available in a small subset of UKB (4366 participants) and were regressed against the available anthropometric and BIA phenotypes. In this model training subset, the imputed DXA (iDXA) phenotypes showed, on average, a 0.81 correlation to the measured DXA and 66% predictive accuracy (Fig. 1a and Supplementary Data 1).

The resultant models were then used to estimate iDXA in the remaining UKB participants and the iDXA phenotypes were used as outcomes in GWAS in the extended UKB cohort (392,535 participants), excluding the individuals that were used to train the iDXA models. This resulted in an effective GWAS sample size of ~ 259,073 participants (i.e., 66% of the total iDXA cohort).

Cumulatively, the iDXA GWAS yielded just under 5000 overlapping genome-wide significant signals, mapping to 1251 quasi-independent loci, at the multiple-testing adjusted threshold of *P* < 1.25 × 10^−8^ (Fig. 1b and Supplementary Fig. 1). Among these, many were well known and had been previously associated with BMI and other obesity traits, such as *FTO*, *MC4R*, and other loci.

At the genome-wide level, the iDXA GWAS were predominantly enriched for genes expressed throughout the different brain regions. This is concordant with previous findings for BMI-associated loci^14^. However, we also observed some enrichment for adipose-, breast- and pituitary-expressed genes (Supplementary Data 2 and Supplementary Fig. 2).

### iDXA signal replication in independent DXA cohorts

This list of identified loci was checked against all known associations in the GWAS catalogue and for associations with BMI and WHR in the UKB cohort (Fig. 1c). After excluding those known signals, the iDXA SNPs mapped to 242 independent loci (Supplementary Data 3). We then sought to replicate some of these signals in the meta-analysis of 4 DXA cohorts, consisting of ~18,000 participants (Supplementary Data 4).

We determined that of the 242 loci identified in the iDXA GWAS, 27 would be replicable within the smaller replication sample size, given the observed iDXA effect sizes. At an FDR of 10%, 6 SNPs replicated successfully in this directly measured and independent DXA cohort, in or near genes *MAFF/PLA2G6, CPS1, ACVR2B/EXOG, FBXO36, ADAMTS14* and *ACADVL/DLG4*. Collectively the variants (19/27) showed evidence of directional consistency between discovery and replication (equivalent to a sign test *P* value of 0.03, Fig. 1d and Supplementary Fig. 3).

To better understand these replicated DXA associations, we also analysed the association patterns they exhibit with the BIA and anthropometric phenotypes used to derive the iDXA traits (Supplementary Data 5 and Supplementary Fig. 4) and also with other iDXA traits (Supplementary Data 6 and Supplementary Fig. 5). Notably, most of these signals appear to be quite pleiotropic and show several associations in the different iDXA GWAS.

We also queried the replicated SNPs for associations with the discovery iDXA traits in the non-white-British participants of UKB (Supplementary Data 7 and Supplementary Fig. 6). While three of the six SNPs showed some concordant evidence of association, most of the ancestry-specific sub-cohort analyses appeared underpowered to confirm or refute the discovered associations due to the smaller sample sizes. It is also important to note that the iDXA phenotypes were derived using the white-British imputation cohort, while body shape and composition have been shown to vary significantly between different ancestries^15^.

### Gene prioritisation at the six replicated loci

The six replicated variants were followed up to establish causal gene candidates within their respective genomic loci, using the GTEx expression quantitative trait loci (eQTL) data^16^. The Approximate Bayes Factor (ABF) posterior probability^17^ and/or the Summary data-based Mendelian Randomization and Heterogeneity in Independent Instruments (SMR and HEIDI) tests^18^ were used to establish pleiotropy between iDXA GWAS signals and the eQTL data.

**Fig. 2:**
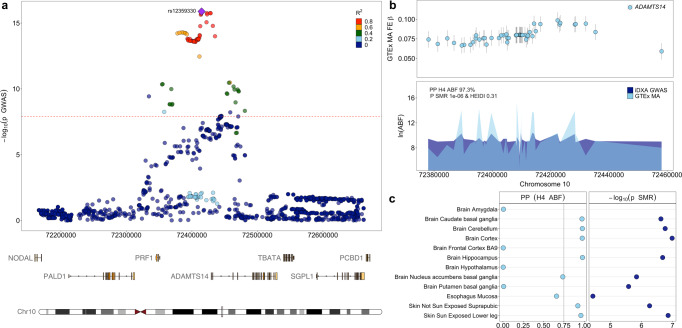
*ADAMTS14* is a strong causal gene candidate in the iDXA GWAS. **a** Association statistics for SNPs within a 1-Mb window of the index SNP, rs12359330-T, on chromosome 10 and their association to the iDXA phenotype. **b** Variants in moderate LD (*R*^2^ > 0.6) with rs12359330-T, were direct eQTLs of *ADAMTS14* within the GTEx tissue-wide fixed-effects (FE) meta-analysis (MA) from up to 714 donors, showing a positive effect on gene expression (upper, shown as beta ± SE) and the eQTL pattern colocalised with the GWAS pattern (lower), with PP H4 ABF of 97.31% and P SMR 10^−6^ (P HEIDI 0.31). **c** Tissue-level colocalisation analyses between the GWAS association pattern in (**a**) and changes in tissue-specific expression of *ADAMTS14* across the GTEx tissues. For the SMR analyses (on the right), all displayed tissues had an FDR-corrected SMR two-sided *P* value < 5% and HEIDI-test *P* value > 5%, indicating pleiotropy at all displayed tissues and for the ABF (on the left) colocalisation was confirmed in tissues where PP H4 ABF is >0.75, thus confirming *ADAMTS14* as a causal candidate gene. Extended data from the SMR and colocalisation analyses can be found in Supplementary Data 8 and 9.

At most of the replicated loci, these analyses implicated multiple causal gene candidates making prioritisation challenging (Supplementary Fig. 7). However, we saw strong evidence of colocalisation between the leg fat-to-lean mass ratio (Leg FMR) GWAS locus at chromosome 10 and *ADAMTS14* eQTLs. The lead variant in the iDXA GWAS at this locus, rs12359330-T, was associated with an increase in leg adiposity and an increase in *ADAMTS14* expression. Specifically, homozygous carriers of rs12359330-T exhibit increased expression of *ADAMTS14* (Fig. 2c) and had 36 more grams of fat on their legs. Thus, the expected effect of null mutations in *ADAMTS14* would be reduced adiposity.

### Null mutations in *Adamts14* confer resistance to weight gain and increased energy expenditure under obesogenic conditions in mice

**Fig. 3:**
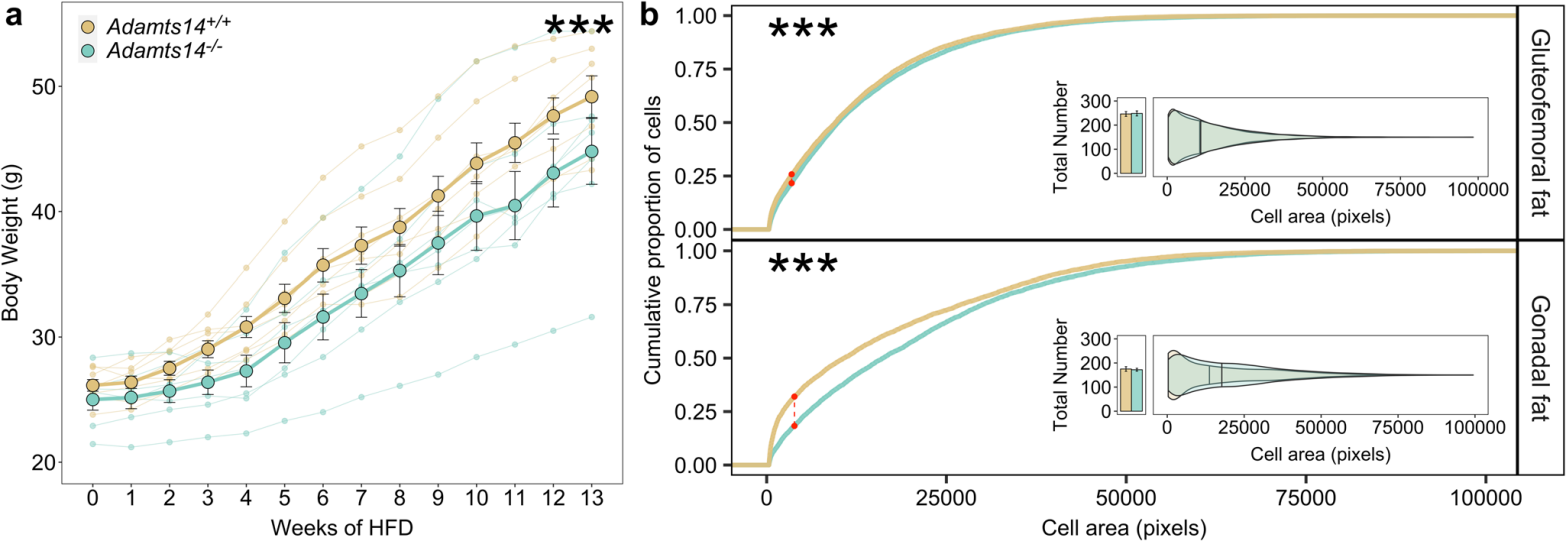
The *Adamts14*^−/−^ mice showed resistance to weight gain and altered adipose histomorphology under HFD conditions. **a** Body weight increase of WT and homozygous-null animals, starting at 2 months old and through the 13 weeks of HFD exposure. Data expressed as mean ± s.e. and analysed in a linear mixed model with repeated measures. *N* = 8 per genotype initially, down to 6 *Adamts14*^*+/+*^ and 7 *Adamts14*^−/−^ by week 13. Extended data can be found in Supplementary Data 11. **b** Cumulative frequency distribution of adipocyte cell-surface area for the gluteofemoral and gonadal fat pads, compared between the two genotypes using a two-sided Kolmogorov–Smirnov test (*P* = 1.376 × 10^−6^ for gluteofemoral fat and <2.2 × 10^−16^ for gonadal fat). The inset shows the total number of cells (expressed as mean ± s.e.) and violin plots for the proportional adipocyte size distributions with the overall means for the two genotypes. *N* = 6 *Adamts14*^*+/+*^ and 7 *Adamts14*^−/−^ and at least 3 independent images quantified per fat pad per animal. Significance denoted as * for *P* < 0.05, ** for *P* < 0.01 and *** for *P* < 0.001. Extended data can be found in Supplementary Data 13.

To test this hypothesis, we obtained mice with a null mutation in *Adamts14*^19^ to assess adiposity phenotypes (Supplementary Fig. 8). *Adamts14*^+/−^ and *Adamts14*^−/−^ animals were viable, bred normally and did not exhibit embryonic lethality. Both genotypes were resistant to weight gain compared to their C57BL/6J littermates over a 13-week period of high-fat diet (HFD) administration (−0.216 ± 0.082 g, *t* = −2.653, *P* = 0.009). This was especially evident in *Adamts14*^+/−^ mice after 6 weeks of HFD administration, with a pronounced divergence in fat mass (5.03 g lower fat mass, *P* = 0.033, Supplementary Data 10 and Supplementary Fig. 9), but not in the *Adamts14*^−/−^ mice after the end of the HFD administration (Supplementary Fig. 10). *Adamts14*^−/−^ mice also had proportionately fewer small adipocytes (Fig. 3b), but adipose morphology was otherwise indistinguishable between the *Adamts14*^+/+^ and *Adamts14*^−/−^ animals and depots of the two groups exhibited comparable proportions of collagen content, as quantified by picrosirius red staining (Supplementary Data 15 and Supplementary Fig. 11).

**Fig. 4:**
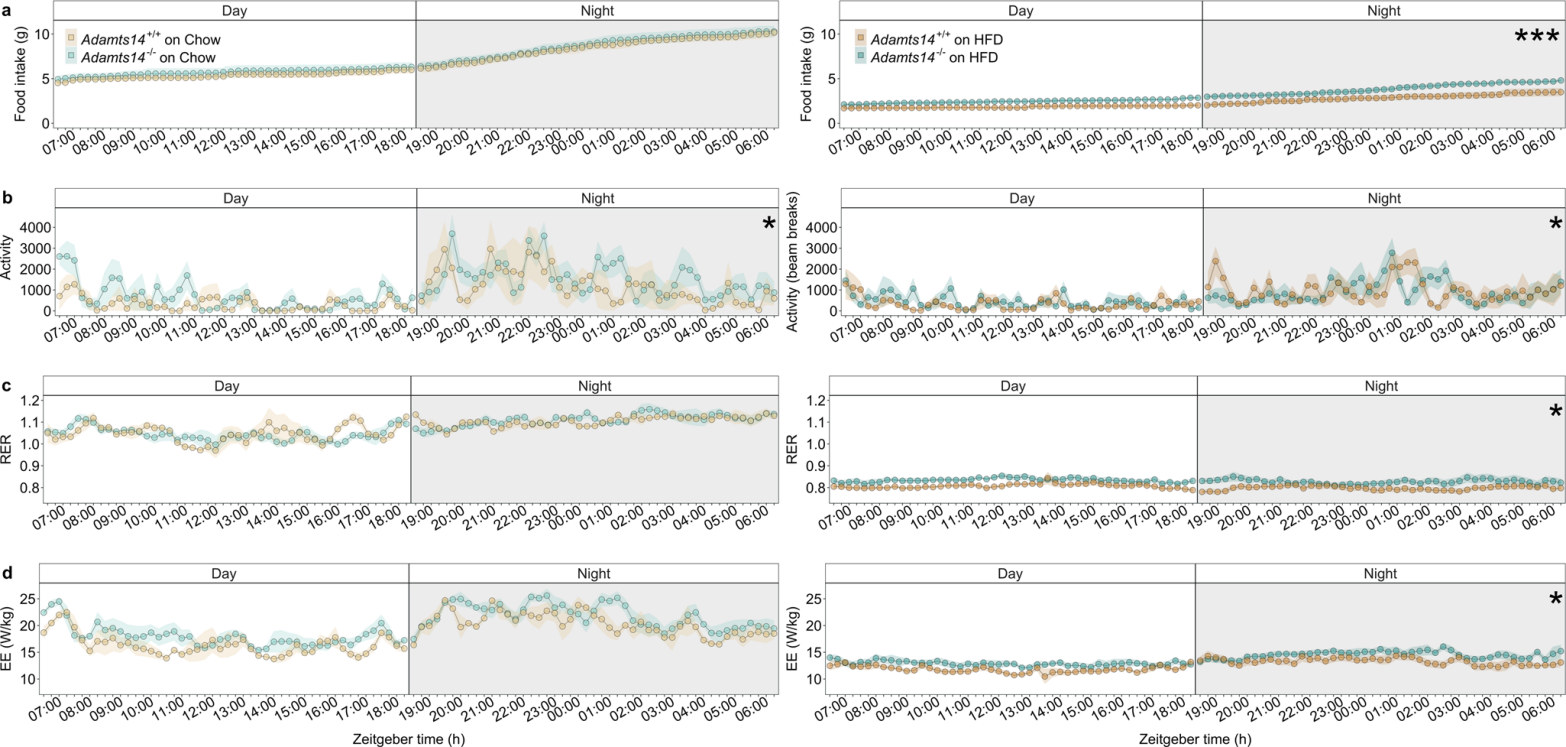
The *Adamts14*^−/−^ mice exhibited hyperphagia and altered energy homoeostasis under normal diet and HFD conditions. Food intake (**a**), activity (**b**), RER (**c**) and energy expenditure (EE, **d**), measured every 15 min over a 24-h time period before (left) and after (right) the 13-week HFD treatment. Data expressed as mean ± s.e. and analysed in a linear mixed model with repeated measures over the 24-h. All tests are two-sided. EE is displayed divided by individual mouse weights, while EE models incorporated mouse weight as a covariate. All models were adjusted for inter-individual mouse and litter variation. For the Chow analyses, *N* = 3 *Adamts14*^*+/+*^ and 5 *Adamts14*^−/−^ (left panels) and *N* = 6 *Adamts14*^*+/+*^ and 7 *Adamts14*^−/−^ for the HFD analyses (right panels). Significance denoted as * for *P* < 0.05, ** for *P* < 0.01 and *** for *P* < 0.001, while the observed test statistics were: food intake on chow (estimate = 0.007, *z* = 1.12, *P* = 0.263) and on HFD (estimate = 0.026, *z* = 9.68, *P* = 3.733x10^−22^); activity on chow (estimate = 1.118, *z* = 2.285, *P* = 0.022), and on HFD (estimate = 0.427, *z* = 2.17, *P* = 0.03); RER on chow (estimate = 0.01, *z* = 1.33, *P* = 0.182) and on HFD (estimate = 0.027, *z* = 2.12, *P* = 0.034); EE on chow (estimate = 0.013, *z* = 1.061, *P* = 0.289) and on HFD (estimate = 0.295, *z* = 2.517, *P* = 0.012). Extended data can be found in Supplementary Data 12.

Homozygous-null mice were also assessed in terms of their energy expenditure (EE), activity and food intake before and after the administration of the HFD (Fig. 4). The *Adamts14*^−/−^ animals consumed more food compared to their WT littermates, particularly after exposure to the HFD (0.026 ± 0.003 g, z = −9.68, *P* = 4x10^−22^). The same pattern was observed with EE, which was significantly increased after the diet treatment (0.295 ± 0.117 W/kg, z = 2.517, *P* = 0.012), while physical activity was higher at both timepoints (1.118 ± 0.489 beam breaks, *t* = −2.285, *P* = 0.022 before HFD and 0.427 ± 0.197 beam breaks, z = −2.17, *P* = 0.03 after). The respiratory exchange ratio (RER) was comparable between the two groups at the beginning of the experiment, but the *Adamts14*^−/−^ mice had significantly higher RER at the end of the experiment (0.027 ± 0.013, *z* = −2.12, *P* = 0.034). Finally, the *Adamts14*^−/−^ animals exhibited shorter tibial (0.23 cm, *P* = 0.008) and gut length (7.5 cm, *P* = 0.009, Supplementary Fig. 10). However, their faecal energy content was unaltered (0.133 ± 0.36 kJ/g, *t* = 0.369, *P* = 0.715, Supplementary Data 14 and Supplementary Fig. 12).

## Discussion

Accurate whole-body and segmental adiposity measures are needed in order to fully delineate the genetic drivers of body composition and its contributions to disease. To this end, several population cohorts have invested in whole-body scans of their participants, such as MRI or DXA scans. GWAS in these cohorts have started to provide some insights into the genetics of human body composition^20–22^. However, these are lagging behind the GWAS signals for traits like BMI and WHR^12,13^, largely due to sample size discrepancies and in turn due to the associated expense. Importantly, the loci emerging from these analyses often show little overlap and thus, viable alternatives are needed to derive detailed adiposity measures.

To this end, we developed a linear model method that successfully imputed DXA measures in UKB, using anthropometric and BIA phenotypes with 66% overall predictive accuracy. This resulted in an effective iDXA sample size of ~259,073, which is almost two orders of magnitude greater than the sample size which was available for the direct DXA measures alone (*n* = 4366). Using the iDXA phenotypes in a GWAS markedly increased our discovery power and led to the identification of hundreds of loci associated with body composition. In an effort to not only replicate the iDXA associations, but also verify the imputation method, we sought replication in the meta-analysis of four separate DXA cohorts. Out of 27 tested signals, 19 were directionally consistent and 6 replicated.

The DXA imputation approach increased our discovery power greatly, however it is important to consider the following issues. Firstly, while the imputation method worked reasonably well for most DXA phenotypes, some predictions were poor (i.e., *R*^2^ ~ 0.25–0.5, Supplementary Data 1). This was predominantly an issue for indices accounting for a lean mass phenotype (i.e., Android LMI, Leg FMR and TLI), which was perhaps to be anticipated, as none of the proxy phenotypes used in the models offer direct measures of lean mass. When using BIA, lean mass is often underestimated in lean subjects and overestimated in subjects with obesity^23,24^, which could help explain the prediction issue.

Secondly, as with any similar approach, there is the concern of whether the genetic signals were associations to the underlying DXA phenotypes or merely to some of the components of the prediction models. This would be impossible to preclude without having a much larger DXA cohort to test or use of out-of-sample validation of the iDXA predictions, which is a limitation of the current study. However, arguably even if that were the case, the resulting associations would still provide knowledge towards human body composition. If accepting the six iDXA loci which were replicated in the meta-analysis of independent DXA cohorts as true DXA associations, we saw that all of them exhibited significant or suggestive associations towards several components of the prediction models (Supplementary Fig. 4), while also associating with several of the iDXA traits (Supplementary Fig. 5). Specifically, some loci appear to be associated with nearly all iDXA traits, regardless of underlying body fat depot. Such loci may affect whole-body composition, while other loci may have more depot-specific effects.

Finally, the six replicated SNPs all had larger effect sizes in the DXA than they did in the iDXA analysis, suggesting that the iDXA GWAS underestimated the effect of the variants on the phenotypic variation, although this would need to be shown in larger DXA samples. While the opposite phenomenon (i.e., inflation of the effect sizes) would have been more problematic, the observed attenuation remains something to consider when using similar methods. Taken altogether this could indicate that, due to study design and limitations surrounding DXA data availability at scale, this study may have picked up on some of the more pleiotropic and obvious associations with human body composition, which are pervasive enough to be detectable through proxy phenotypes and in smaller sample sizes.

We followed up the six replicated loci with colocalisation analyses using eQTL data. These showed several candidate causal genes at most loci (Supplementary Fig. 7), but clearly highlighted *ADAMTS14* as the putative causal candidate gene at the chromosome 10 locus surrounding signal rs12359330 (Fig. 2). Specifically, colocalisation analyses showed that variants associating with a decrease in expression of *ADAMTS14* also associated with decreased fat mass. Subsequent to the selection of *ADAMTS14* for further follow-up, Johansson and colleagues conducted GWAS on the same cohort, focusing on BIA-based segmental adiposity^25^. In doing so they identified 29 novel associations, many of which were highlighted in this work, *ADAMTS14* inclusive.

To better understand this genetic association, we obtained *Adamts14*-null mice and characterised their metabolic and physiological response to an obesogenic diet intervention. While we used a limited number of mice which reduced our statistical power, the effects of *Adamts14* gene knockout were sufficiently large to reveal significant and consistently reduced adiposity, weight gain, hyperphagia and increased energy expenditure, alongside adipocyte hypertrophy (Figs. 3 and 4). Specifically, by the end of the HFD exposure the *Adamts14*^−/−^ mice weighed on average 4.5 g less than their WT littermates despite consuming, on average, 1.3 g more food daily. The resistance to weight gain in *Adamts14*^−/−^ mice was explained, at least in part, by their higher energy expenditure (Fig. 4). Their RER, which is an index of carbohydrate versus lipid fuel utilisation, diverged after exposure to HFD, such that the expected RER suppression with HFD was less pronounced in *Adamts14*^−/−^ animals, indicative of maintained metabolic health. They also exhibited shorter tibial and gut lengths, but similar nasoanal lengths. As the latter is the most commonly used measure of murine body size^26^, a more specialised phenotype, i.e., a bone or cartilage phenotype could explain the reduced tibial length. Gut length has been shown to fluctuate under different dietary compositions and availabilities^27^ and could have an impact on the efficiency of dietary fat assimilation and adiposity. However, the increased RER on the HFD suggests a greater reliance on carbohydrate oxidation and we observed no direct evidence of impaired nutrient resorption on the lipid-dense diet (Supplementary Fig. 12). Impaired nutrition would also arguably be contradictory towards the observed adipose hypertrophy.

*Adamts14* encodes a metalloproteinase and aminoprocollagen peptidase and takes part in the maturation of type-I collagen fibres^28^. Metalloproteinases are known regulators of body composition and, of direct relevance^29–32^, in the absence of certain metalloproteinases, adipogenesis is halted due to impaired degradation of the extracellular matrix (ECM), which becomes dense and fibrotic, hindering the hypertrophic expansion of adipocytes. Since *Adamts14* plays a role in the deposition, as opposed to degradation, of ECM components, it could exert the opposite effect. However, we did not observe a genotype effect when quantifying the fibrillar collagen in the adipose tissue of the *Adamts14*^+/+^ and *Adamts14*^−/−^ animals. This indicates that the observed adipose effect could be mediated via change in the availability of other *Adamts14* substrates. Notably, Dupont et al.^33^ recently showed that ADAMTS14 was an efficient activator of VEGFC signalling, and its absence caused altered lymphangiogenesis in mice. As elevated VEGFC is associated with obesity and overexpression of *Vegfc* promotes fat mass gain and insulin resistance^34,35^, the reduced weight gain and improved metabolic profile we observed in the *Adamts14*^−/−^ mice, could be caused via a reduction of VEGFC levels, concordant with other published models^34,35^. Other ADAMTS14 substrates include members of the TGF-β receptor signalling pathway^36^ which are known regulators of adipogenesis^37,38^, offering alternate explanations for the adipocyte hypertrophy observed in the *Adamts14*-null mice. While more experimental work at a larger scale is needed to pinpoint the exact molecular mechanism linking reduced weight gain and altered adipocyte size with improved metabolic rate and health in the *Adamts14*^−/−^ mice, these results confirm directionality and conservation of effect across mammalian species.

## Methods

### Human cohort data

#### The UKB cohort

The UKB is a large population-based cohort with over 500,000 participants, aged 40–69, which were recruited in the UK over a period of 5 years, from 2006 to 2010. Its aim is to improve the prevention, diagnosis and treatment of serious and life-threatening illnesses affecting people of middle- and old-age. UKB includes extensive phenotypic and genotypic data on its participants, including questionnaire data, physical measures, blood and urine sample assays, accelerometry, multimodal imaging, genome-wide genotyping and longitudinal follow-up^39^. All participants gave written informed consent, and the study was approved by the North West Multicentre Research Ethics Committee.

Relevant to this study, participants underwent anthropometric examinations of their height, weight, waist and hip circumference and BIA on a Tanita BC418MA body composition analyser. These measurements were available for the entirety of the cohort (493,088 participants). Participants’ body composition was further assessed using the GE Lunar iDXA scanner, for a subset of 5170 individuals. Scans of the whole body are analysed by the radiographer at acquisition to generate all numerical measures of bone mass and body composition. These measures are transferred directly from the instrument to UKB servers and require no post-processing.

UKB participants were genotyped under two Affymetrix arrays, which show a 96% SNP overlap and resulted in 820,967 genetic markers being genotyped. SNPs were excluded on the basis of missingness and departure from Hardy–Weinberg equilibrium (HWE)^40^. Imputation was performed by UKB, using SHAPEIT2 and IMPUTE2 on the UK10K, HRC and 1000 Genomes Phase 3 reference panels^39^.

Phenotypes and genotypes used within this work were directly downloaded from the UKB website, under application 19655.

#### ORCADES

The Orkney Complex Disease Study—ORCADES cohort is a population-based isolate that includes 2215 individuals. Its aim is to characterise the genetic and epidemiological components that underlie quantitative traits and diseases in the Orkney Islands of Scotland. Individuals of all ages were recruited based on the basis of their Orcadian heritage and have at least two Orcadian grandparents, thus maintaining the homogeneous genetic background of the cohort. Data collection was carried out between 2005 and 2011 in Orkney by trained research nurses. The Orkney Research Ethics Committee and North of Scotland Local Research Ethics Committee granted the study ethical approval and all participants individuals gave written, informed consent prior to participating in any research, such as broad-ranging health and disease or population research, including biobanking of samples or record linkage to hospital admissions or to other records^41^.

A subset of 1256 individuals of the ORCADES cohort underwent full body composition analysis on the Hologic fan beam DXA scanner (GE Healthcare). Trained radiology research nurses generated the scans and ensured the correct positioning of the participants’ pelvis, arms and legs. The APEX2 software was used to generate individual measurements for bone, lean, and fat tissue of the head, arms, trunk, legs and total body. Subsequently, the APEX4 software was used to estimate the individuals’ android, gynoid and visceral fat and lean mass content by others and used in the context of this project.

DNA was extracted from whole blood and genotyped on three different SNP chips: Illumina Infinium Human Hap300v2, OMNI1 and OMNI Express. Each covers between ~300,000 and 1,000,000 variants across the genome, only ~160,000 of which overlap between the chips. Genotypes were called via the Illumina BeadStudio and GenomeStudio software. Samples or SNPs with a call rate of under 97%, samples with a gender mismatch and SNPs deviating from HWE with a *P* value of smaller than 1e-6 were excluded from all further analyses. Imputation was done separately for the Hap and Omni chips, using the IMPUTE software (version 2.2.2) on the 1000 Genomes European imputation panel and the exomes of 90 Orcadians. The two imputations were then merged, covering 37.5 million polymorphisms across the genome^42^.

In the context of this work, we used SNP-phenotype associations for the 27 SNPs outlined in Table 1, resulting from 1253 total ORCADES participants.

**Table 1:** SNP information for the 27 prioritised loci in Discovery (iDXA) and Replication (DXA MA)

rsid	Phenotype	Gene	chr_pos	a1	a0	freq1	iDXA—Discovery			DXA MA—Replication		
							beta1	s.e.	*P* value	beta1	s.e.	*Q*-value
**rs4820325**	**Leg FMR**	***MAFF/PLA2G6***	**22_38599978**	**A**	**G**	**0.58**	**−0.022**	**0.002**	**9.61E-23**	**−0.043**	**0.011**	**9.55E-04**
rs117068593	Leg FMR	*RIN3*	14_93118229	T	C	0.19	−0.026	0.003	7.25E-20	−0.024	0.014	0.125
**rs1047891**	**Android LMI**	***CPS1***	**2_211540507**	**A**	**C**	**0.32**	**0.022**	**0.002**	**1.31E-19**	**0.028**	**0.011**	**4.65E-02**
rs150985439	Android LMI	*CYBRD1*	2_172417398	C	G	0.25	−0.023	0.003	3.50E-19	0.006	0.012	0.781
rs62218301	Leg FMR	*LOC100506403*	21_36741262	G	A	0.17	−0.027	0.003	3.69E-19	−0.006	0.014	0.517
**rs13072731**	**Android LMI**	***ACVR2B/EXOG***	**3_38533335**	**C**	**A**	**0.61**	**0.021**	**0.002**	**5.15E-19**	**0.022**	**0.011**	**8.34E-02**
rs17684944	Leg FMR	*NMT1/MIR6784*	17_43187141	C	T	0.55	0.02	0.002	9.80E-19	0.013	0.011	0.216
rs2736208	Gynoid fat mass	*TRPS1*	8_116826244	C	T	0.76	0.023	0.003	9.83E-19	0.003	0.012	0.584
rs11079849	SFI	*IGF2BP1*	17_47090785	T	C	0.33	−0.02	0.002	2.33E-17	−0.015	0.011	0.216
**rs17325374**	**Android FMI**	***FBXO36***	**2_230822858**	**G**	**A**	**0.32**	**0.02**	**0.002**	**3.58E-17**	**0.033**	**0.011**	**1.88E-02**
rs3749748	Android LMI	*LINC01184*	5_127350549	T	C	0.25	0.022	0.003	6.00E-17	−0.001	0.012	0.691
**rs12359330**	**Leg FMR**	***ADAMTS14***	**10_72414845**	**T**	**C**	**0.27**	**0.021**	**0.003**	**1.32E-16**	**0.027**	**0.012**	**6.86E-02**
rs12451882	Total lean %	*UBTF*	17_42301922	T	C	0.31	−0.02	0.002	2.06E-16	−0.005	0.011	0.517
rs4527444	Android LMI	*PCDH7*	4_30842780	G	A	0.54	0.019	0.002	3.20E-16	−0.007	0.011	0.781
rs1504797	Android LMI	*MMP16*	8_89434405	C	T	0.3	−0.02	0.002	9.00E-16	−0.008	0.012	0.485
rs2881198	Android LMI	*LOC730338*	7_46634506	C	G	0.54	−0.018	0.002	1.82E-15	−0.019	0.011	0.125
rs10761129	Android LMI	*ROR2*	9_94486321	T	C	0.67	0.019	0.002	3.34E-15	0.002	0.011	0.600
**rs2242449**	**Total lean %**	***ACADVL/DLG4***	**17_7095507**	**T**	**C**	**0.43**	**−0.018**	**0.002**	**6.94E-15**	**−0.035**	**0.011**	**7.40E-03**
rs4832298	Leg FMI	*CHMP3*	2_86764004	T	C	0.69	−0.019	0.002	1.10E-14	0.006	0.011	0.781
rs7893571	Android fat mass	*RSU1*	10_16750129	T	G	0.66	0.018	0.002	1.51E-14	−0.008	0.012	0.781
rs11525873	Android LMI	*ZC3HAV1/TTC26*	7_138817193	C	T	0.1	−0.029	0.004	2.88E-14	−0.011	0.018	0.493
rs6557791	Leg FMR	*FAM160B2*	8_21949663	C	T	0.6	−0.018	0.002	3.44E-14	0.001	0.011	0.691
rs12274114	Android LMI	*LRP5/PPP6R3*	11_68255577	C	A	0.28	−0.019	0.003	5.18E-14	−0.023	0.012	0.102
rs1441264	SFI	*LINC00331*	13_79580919	A	G	0.59	0.018	0.002	6.02E-14	−0.013	0.011	0.875
rs2504236	Android LMI	*FLT3*	13_28613298	T	C	0.59	−0.017	0.002	1.11E-13	−0.014	0.011	0.216
rs4660586	Gynoid fat mass	*HIVEP3*	1_42407229	T	C	0.74	−0.019	0.003	1.82E-13	0.005	0.012	0.781
rs59893724	SAT	*SSBP2*	5_80830788	G	A	0.25	−0.019	0.003	6.00E-13	−0.019	0.012	0.182

a1 denotes the effect allele and freq1 is the frequency of the effect allele in Discovery, while a0 is the other allele. beta1 is the effect on the Phenotype per copy of a1 and s.e. is the error of beta1. Discovery presented as two-sided *P* values and Replication presented as FDR-corrected one-sided *Q*-values and considered successful if *Q*-value < 0.1 (in bold). Phenotype calculations and explanations can be found in Supplementary Data 1, but briefly FMR indicates fat-to-lean mass ratio, LMI indicates lean mass index and SFI is the segmental fat index. Replication cohort summary statistics are given in Supplementary Data 4. Gene column was annotated simply by proximity to the lead-associated variant (±500 kb). chr_pos denotes the chromosome and chromosomal location of each variant. Genomic locations and annotations given in GRCh37/hg19 and dbSNP build 150.

#### EPIC-Norfolk

The European Prospective Investigation of Cancer (EPIC) is a large multicentre prospective cohort study that focuses on the connection between diet, lifestyle factors and cancer. The Norfolk cohort includes 25,639 individuals, aged 40–79, living in Norwich and the surrounding towns and rural areas. The participants were recruited between 1993 and 1997 and have been contributing information about their lifestyle and health through questionnaires and health checks for over two decades. All participants gave signed informed consent and The Norwich District Health Authority Ethics Committee approved the study^43^.

A subset of the cohort was DXA scanned at the fourth health check, 20 years after initial recruitment, at the EPIC-Norfolk Unit at the Norwich Community Hospital. The Lunar Prodigy advanced fan beam scanner (GE Healthcare) and the enCORE software version 14.10.022 (GE Healthcare) were used. Participants were scanned by trained operators, using standard imaging and positioning protocols. The enCORE software was used to demarcate the regional boundaries. All the images were manually processed by one trained researcher, who corrected demarcations according to a standardised procedure^44^.

The EPIC samples were genotyped using Affymetrix UK Biobank Axiom Array and genotypes were called using Axiom GT1. SNPs and samples were subject to the following quality control (QC) criteria: Hardy–Weinberg *P* value > 10e-6, MAF >0%, sample and SNP call rate ≥95%, and samples’ heterozygosity check and gender check. Imputation was performed on Haplotype Reference Consortium r1.0/r1.1 and the UK10K plus 1000 Genomes phase 3 reference panels via IMPUTE4 software and via Sanger Imputation Service, and all SNPs with an info-score ≥0.4 were kept^45^.

In the context of this work, we used SNP-phenotype associations for the 27 SNPs outlined in Table 1, resulting from 4134 total EPIC-Norfolk participants.

#### Fenland

The Fenland Study is an ongoing population-based cohort study, that includes 12,435 adults aged 29–65 years in Cambridgeshire, UK. The first phase of the study investigates the interaction between environmental and genetic factors determining obesity, T2D, and related metabolic disorders. Volunteers were recruited from general practice registers between 2005 and 2015. A follow-up study (Phase 2) was launched in 2014 with the objective of studying the relationship between change in objectively quantified behaviours and body composition and metabolic risk. The Fenland study was approved by the Cambridge Local Research Ethics Committee and all participants gave written informed consent^46^.

Body composition by DXA was assessed, using the Lunar Prodigy Advanced fan beam scanner (GE Healthcare) with a constant pixel size of 1.2 mm. Estimates of total body fat mass and total abdominal fat (g) were derived with Prodigy enCORE software (version 10.51.006; GE Healthcare). The DXA abdominal fat region (g) was defined by quadrilateral boxes with the base of the box touching the pelvis and the lateral boundaries extending to the edge of the abdominal soft tissue^46,47^.

Participants were genotyped on the Affymetrix UK Biobank Axiom Array, and genotypes were called using Axiom GT1. SNPs and samples inclusion criteria were Hardy–Weinberg *P* value > 10e-6, MAF >0%, sample and SNP call rate ≥95%, and samples’ heterozygosity check and gender check. Imputation was performed on Haplotype Reference Consortium r1.0/r1.1 and the UK10K plus 1000 Genomes phase 3 reference panels via IMPUTE4 software and via Sanger Imputation Service, and all SNPs with an info-score ≥0.4 were kept^45^.

In the context of this work, we used SNP-phenotype associations for the 27 SNPs outlined in Table 1, resulting from 8034 total Fenland participants.

### Phenotype imputation

In March 2018, derived DXA phenotypes were available for a subset of 5170 individuals within the UKB study. These phenotypes included fat mass, lean mass, bone mass and total mass, for the following seven general body areas: android, gynoid, arm, leg, trunk, VAT and total body. Phenotypes were downloaded and used for the purposes of this work, alongside anthropometric (height, weight, waist and hip circumference), BIA (fat, fat-free and total mass for the arms, legs, trunk and total body, basal metabolic rate and total water weight) and demographic phenotypes (sex, age, assessment centre, Townsend deprivation index, educational attainment, Northing and Easting, genetic ethnicity). For the physical measures, where these phenotypes had been recorded at multiple instances, the measurements were averaged. Related and non-white-British individuals were excluded from all analyses, based on fields 22011 and 22006.

After exclusions, the remaining UKB participants were separated into two sub-cohorts; the UKB DXA cohort (4,366 participants), used to impute the DXA phenotypes (iDXA) onto the UKB iDXA cohort (392,535 participants), which was used for subsequent genetic discovery analyses. The UKB DXA cohort was also part of the DXA replication meta-analysis (MA) cohort. Apart from the derived DXA phenotypes, we also created composite phenotypes and indices to approximate adipose distribution patterns (calculated as outlined in Supplementary Data 1).

DXA phenotypes were sex-separated and imputed, using linear models that incorporated all of the available anthropometric and BIA phenotypes, as mentioned above and seen below,

$${{{\rm{DXA}}}}=\beta 0+\beta 1\times 1+\beta 2\times 2+\beta 3\times 3+\ldots+\beta {{{\rm{n}}}}\times {{{\rm{n}}}}+\varepsilon$$

where, *β* is the effect of each x phenotype on the measured DXA, *β*0 is the intercept of the linear model, *ε* is the normally distributed residual error and the proxies (x) used were Age, Height, Waist circumference, Hip circumference, WHR, Weight, BMI, BIA total fat %, BIA total fat mass, BIA total fat-free mass, BIA total water mass, BIA basal metabolic rate, BIA trunk fat %, BIA trunk fat mass, BIA trunk fat-free mass, BIA total trunk mass, BIA leg fat %, BIA leg fat mass, BIA leg fat-free mass, BIA leg total mass, BIA arm fat %, BIA arm fat mass, BIA arm fat-free mass and BIA arm total mass.

Effect estimates for each of these model components were then used to estimate the iDXA phenotypes in the individuals which had anthropometric and BIA phenotypes measured but lacked the DXA ones. To assess imputation efficacy, the iDXA phenotypes were correlated to the original DXA ones.

### GWAS methodology

All of the sex-separated iDXA phenotypes were natural log-transformed to achieve a normal distribution, prior to GWAS. Following that, iDXA values lying further than six standard deviations on either side of the population mean were removed. They were corrected for age, assessment centre, geographical coordinates, Townsend deprivation index, educational attainment, the first 20 principal components (PCs), genotyping array and batch, as is standard practise and as seen below. QQ plots were visually inspected to confirm that the covariates used to adjust for population stratification were sufficient to control type-I error rate.

$$\begin{array}{c}{iDXA}\,{phenotype}\,{ \sim }\,{Age}+{Assessment}\,{centre}+{Northing}\\+{Easting}+{Townsend}\,{deprivation}\,{index}+{Educational}\,{attainment}\\+{Genotyping}\,{batch}+{Genotyping}\,{array}+{PC1}+\ldots+{PC}20\end{array}$$

The resulting residuals were inverse rank transformed and values lying four standard deviations at either side of the population mean were removed. Residuals from males and females were merged and used for GWAS. For each SNP, the iDXA phenotypes were regressed on the three possible genotypes using REGSCAN (v0.5)^48^ and assuming additive genetic effects. SNPs with a MAF < 0.001 and an imputation quality score <0.4 were excluded from analyses.

To correct for multiple testing, the R package PhenoSpD (v1.0.0)^49^, was used to ascertain the effective number of tests conducted, given the phenotypic similarity of the iDXA phenotypes, which was four. The genome-wide significance threshold was then adjusted accordingly and set at 1.25 × 10^−8^.

SNP associations below this threshold were compiled across all iDXA phenotypes, sorted into quasi-independent signals based on their location (i.e., signals lying more that 1 Mb apart were considered independent) and assessed for novelty, checking for previous associations with any adiposity or anthropometry phenotypes in the GWAS Catalogue, under the keyword obesity (accessed April 2018, as seen in Supplementary Data 16—an updated version can also be found in Supplementary Data 17)^50^. SNPs within published loci and SNPs which were associated with BMI or WHR in UKB (*P* value < 10^−12^) were excluded. The remaining associations were manually inspected to exclude residual known or linked loci.

A tissue enrichment analysis was also performed on the iDXA GWAS, using LD score regression applied to specifically expressed genes (LDSC-SEG v1.0.1^51^) and tissue-specific annotations from GTEx^52^, accessed via https://github.com/bulik/ldsc/wiki/Cell-type-specific-analyses. Results were FDR-corrected for the number of tested tissues, multiplied by the effective number of GWAS traits.

### Replication

Independent novel loci were followed up with a replication power calculation assuming the effect sizes in the iDXA GWAS would be equal to those in the DXA analyses, as follows:

$${{se}}_{{DXA}}={\sqrt{2{\times N}_{{DXA}}\times {{freq}1}_{{iDXA}}\times \left(1-{{freq}1}_{{iDXA}}\right)}}^{-1}$$

$${p}_{{DXA}}={Normal}\left(\left|{\beta }_{{iDXA}}/{{se}}_{{DXA}}\right|, 0 \,,\,1\right)$$

Where *freq1*_*iDXA*_ is the frequency of the effect allele and *β*_*iDXA*_ is the effect estimate per copy of that allele, as observed in the iDXA Discovery, and *N*_*DXA*_ corresponds to ~18,000 DXA participants from the EPIC-Norfolk, Fenland, UKB DXA and ORCADES studies.

Genotype and DXA phenotype information were submitted to analysts from EPIC-Norfolk and Fenland as outlined in Table 1 and Supplementary Data 1. We conducted the UKB DXA and ORCADES analyses in the same manner. The DXA phenotypes were transformed in the same way the iDXA were and were used to test for association with allelic dosage for the selected 27 iDXA SNPs, in a total replication cohort size of 17,787 DXA participants. Replication was considered successful when the direction of effect was consistent with the one observed in iDXA and the DXA one-sided *P* value was < 0.1 after FDR correction. A binomial test was used as a sign test over all 27 iDXA SNPs.

Replicated signals were looked-up within the UKB non-white-British participants, for the corresponding iDXA phenotypes. Individuals were grouped into three further broad ancestry groups using data from field 21000, as follows; Other white (*n* = 29,015) comprising individuals not included in the Discovery analyses who were ethnically white, Irish or of Any other white background, Asian (*n* = 10,920) including Chinese, Indian, Bangladeshi, Pakistani, Any other Asian background and Asian or Asian British individuals and Black (*n* = 7644) consisting of African, Any other Black background, Black or Black British and Caribbean participants. For participants within each of these ancestry groups, genotypes at the replicated loci were extracted using qctool (v2.0.6) and regressed against the corresponding Discovery iDXA trait in a linear mixed model. To do this, fixed effects of SNP dosage and ancestry group were fit alongside a random effect of ethnicity (field 21000). Effect estimates were aligned towards the discovery effect alleles.

Replicated signals were further looked-up for associations with the anthropometric and BIA phenotypes used in the iDXA models, using UKB summary statistics from ref. ^53^ accessed via Phenoscanner^54^.

### Causal gene identification

Tests of colocalisation between the iDXA GWAS and gene expression data were conducted, using Summary data-based Mendelian Randomisation and Heterogeneity in Independent Instruments (SMR-HEIDI, version 0.68^18^) and the Approximate Bayes Factor (ABF) method in the R package coloc (version 3.2-1^17^). Analyses were performed on either the GTEx multi-tissue meta-analysis statistics or the tissue-specific data (both available via https://gtexportal.org for V7^16^) and using the fixed-effects summary statistics for the former. Statistical thresholds for colocalisation were set at an FDR-corrected SMR *P* value < 5% and HEIDI-test *P* value > 5% and at PP H4 ABF was >75%, in accordance with others^55–57^ and in these cases it was deemed that there is sufficient evidence for a shared variant to drive the changes in gene expression and in the GWAS phenotype.

### Mouse husbandry

The *Adamts14*^−/−^ mice were created as described in^19^ and obtained in collaboration with the Colige group at the University of Liege. Briefly, a genetrap cassette was inserted between exons 4 and 5 in ES cells. Clones were fused to C57bl/6 blastocysts to obtain mosaics that were crossed to give homozygous mice. The protein was not detectable in embryonic or adult skin in homozygous mice. Sperm from heterozygous (*Adamts14*^+/−^) C57BL/6J animals was shipped to Edinburgh frozen and used to perform in vitro fertilisation (IVF) to C57BL/6J females, at the BVS Transgenic Core facility. To do this, ten embryos were transferred per oviduct of four female mice. All mice became pregnant and subsequently produced 37 pups. Resulting heterozygous animals were initially metabolically characterised and also bred to create colonies of *Adamts14*^−/−^ and *Adamts14*^+/+^ animals.

All experiments were performed under PPL 60/8117, appropriate PILs granted under the Home Office Scientific Procedures (Animals) Act 1983 and after full ethical review by the University of Edinburgh Biological Sciences Services. Male mice were used for all experiments and were maintained single-housed in either standard or individually ventilated cages with ad libitum access to food (CRM E, Special Diets Services) and water at the Little France BRR facility. They were kept at 19–22 °C and maintained with a 12-h light/dark cycle with lights on at 7 am.

### Mouse genotyping

Genomic DNA from all live-born mice was extracted from ear tissues and used to genotype by PCR for the targeted alterations. DNA extraction was performed via the digestion of the ear tissues in 20 μl DNAreleasy (1:3 dilution in water, Anachem, UK) at 75 °C for 5 min, followed by 95 °C for 2 min. In total, 1–2 μl of DNA were used as a template per PCR reaction. PCR reactions that specifically amplify the targeted alteration were designed. Primers used were: GFP_F_: 5′-AGCTGGACGGCGACGTAAAC-3′ with GFP_R_: 5′-GCGCTTCTCGTTGGGGTCTT-3′ and F: 5′-GTTCAGTGGGGAGTGAGCCATTAA-3′ with WT_R_: 5′-CTGTGTGCTTGCTGTGATGGCTG-3′ or KO_R_: 5′-CCTGGACCAGCTGTGATGGCTG-3′. The first reaction amplified part of the GFP sequence within the inserted cassette and yielded a 596-bp-sized amplicon. The second reaction spanned the break site (where the cassette was inserted) and yielded a 252 or 251-bp sized amplicon. Both assays were run as follows: 95 °C for 5 min, 30 cycles of 95 °C for 30 s, 65 °C for 45 s and 72 °C for 45 s, then 72 °C for 5 min and according to the Platinum™ SuperFi™ PCR Master Mix (Thermofisher, 12358250) suggested concentrations.

### In vivo metabolic phenotyping

At the age of approximately two (for *Adamts14*^−/−^ mice) or three (for *Adamts14*^+/−^ mice) months, the mice were subject to a 58% high-fat diet (Research Diets Inc, 12331) for up to 13 weeks by allowing the animals ad libitum access to the diet. Our experimental design included the initial cohort of *Adamts14*^+/−^ and *Adamts14*^+/+^ mice, which comprised of five mice per group (and 2 litters per group), the 12-week cohort of *Adamts14*^−/−^ and *Adamts14*^+/+^ mice, with groups of 10 (7 litters) and 8 mice (5 litters) accordingly and finally the 6-week cohort of *Adamts14*^−/−^ and *Adamts14*^+/+^ mice, in groups of 10 (2 litters) and 8 (1 litter) over a 6-week period. This is also represented in Supplementary Fig. 8.

Animals were weighed weekly and had their lean and fat mass determined by time domain-nuclear magnetic resonance (TD-NMR, Bruker) at multiple timepoints throughout each experiment.

PhenoMaster cages (TSE systems, Germany) were used to assess energy expenditure, locomotor activity and food and drink intake of the mice at the beginning and end of the experimental period. To do this, mice were placed in individual monitored cages with ad libitum access to food and water. The animals were allowed to acclimate to their environment for 24 h before the collection of experimental data. Measurements for each parameter were taken continuously for a period of at least 24 h and were recorded every 15 min.

oGTTs were carried out at least at the beginning and end of the HFD treatments. On the morning of the oGTT, the animals were fasted for 5 h by removing their food and transferring them to fresh cages, with ad libitum access to water. A 20% glucose solution in water was ingested by oral gavage at a concentration of 2 mg glucose per gram of body weight. Blood glucose levels were measured using a glucometer (OneTouch Ultra, LifeScan), prior to glucose administration and after at 15-, 30-, 60- and 120-min intervals from a small drop of blood from a tail-nick.

At the end of experiments, animals were sacrificed humanely, by rising CO_2_ concentration and death was confirmed by cervical dislocation. Mice were also culled in accordance with the project license, if they appeared unwell, i.e., exhibited a significance weight loss, absence of grooming, etc. Measurements were removed from experiments insofar as they were affected by the reason a mouse failed to reach the experimental endpoint. Tissues were harvested and immediately frozen under dry ice and stored at −80 °C or collected in 4% paraformaldehyde (PFA) and then dehydrated for at least 24 h in 75% ethanol.

### Adipose histomorphometry and collagen quantification

Fixed and dehydrated adipose tissue was embedded in paraffin and sectioned. For collagen quantification, 5-μm sections were stained with Picro Sirius Red (PSR) (Abcam, ab150681). Mounted sections were viewed with brightfield microscopy. Histomorphometry of adipocytes from the gonadal and gluteofemoral depots was assessed using the Adiposoft plugin in ImageJ. To quantify fibrillar collagen, a pixel classifier was trained in QuPath 0.3.2 using RTrees with ‘gaussian’ and ‘weighted deviation’ features selected at ‘Full’ resolution^58^. Pixels in each image were classified as one of ‘psr_positive’, ‘psr_negative_cellular’, and ‘fat’ and a categorical classified .tiff saved as an output along with the number and percentage of pixels of each class. The percentage of ‘psr_positive’ across the total image area was then used to indicate the amount of fibrillar collagen per section.

### Faecal bomb calorimetry

At the end of the HFD exposure period, faecal samples were collected from the individual mouse cages, alongside a diet sample, and stored at −80 °C until ready for measurement. To measure the energy content of the food and faeces, samples were weighed before and after drying at 60 °C (Gallenkamp Oven) for 14 days until weight stable (Ohaus balance 4 d.p.). This enabled the water content to be calculated. They were then homogenised in a blender, compressed into one or two carefully weighed pellets of 0.15–0.25 g (weighed to 4 d.p.) per mouse and combusted in a Bomb calorimeter (Parr 6100 calorimeter using a 1109 semi-micro bomb). The machine was calibrated daily using pellets of Benzoic Acid.

### Statistical analysis of mouse data

All statistical analyses of mouse data were carried out in R (4.0.1) statistical environment^59^. Comparisons between genotype groups were performed using Two-Sample t-tests for analyses at singular timepoints and linear mixed models, accounting for the individual animal and litter variation, for analyses over multiple timepoints, using glmmTMB (v1.0.2.1). For the indirect calorimetry experiments, the models were also adjusted for phase (day/night) to account for nocturnal behaviours and in cases where the response variable was a count distribution (i.e., for activity counts), a Poisson error structure was used. The cumulative frequency distribution of adipocyte sizes in different genotype groups was compared using a Kolmogorov–Smirnov test. Mixed models were used for the comparison of fibrosis levels in different tissues for the two genotype groups and included a random effect for individual mice and litter. The area under the curve (AUC) was additionally calculated to compare glucose levels between the two genotypes, using the AUC function in the DescTools package^60^.

### Reporting summary

Further information on research design is available in the Nature Portfolio Reporting Summary linked to this article.

## Supplementary information

Supplementary Information

Description of Additional Supplementary Files

Supplementary Data 1-17

Reporting Summary

## Data Availability

This research has been conducted using the UK Biobank Resource, approved under application 19655. Processed cross-tissue and tissue-wide GTEx data are available on the GTEx portal (https://gtexportal.org). GWAS summary statistics are available from the University of Edinburgh’s DataShare repository (10.7488/ds/2973). All other data supporting the findings of this study are available through the supplement.
