# Supplementary material for: Large scale phenotype imputation and in vivo functional validation implicate ADAMTS14 as an adiposity gene
Source: Nat Commun. 2023 Jan 19;14:307. doi: 10.1038/s41467-022-35563-0 (PMC9852585; doi:10.1038/s41467-022-35563-0)

Supplementary Figures

Figure S1 | Manhattan and QQ plots for the iDXA GWAS. 2

Figure S2 | iDXA GWAS tissue enrichment. 4

Figure S3 | Six out of 27 prioritised loci replicated in the DXA cohort MA at a 10% FDR. 5

Figure S4 | Lookup of replicated signals in the BIA and anthropometric components of the iDXA imputation models. 6

Figure S5 | Pleiotropic iDXA associations 7

Figure S6 | Lookup of replicated signals in other ancestry groups 8

Figure S7 | Multiple causal gene candidates at most replicated loci. 9

Figure S8 | Experimental design for the phenotyping of the *Adamts14*$^{+/-}$ and *Adamts14*$^{-/-}$ mice. 10

Figure S9 | The *Adamts14*$^{+/-}$ mouse was resistant to weight and fat gain and had improved glucose homeostasis under HFD conditions 11

Figure S10 | The *Adamts14*$^{-/-}$ mouse shows no differences in adiposity and glucose homeostasis 12

Figure S11 | No histomorphological difference seen between *Adamts14*$^{-/-}$ and *Adamts14*$^{+/+}$ mice. 13

Figure S12 | No apparent differences in nutrient absorption after 6 weeks of HFD exposure. 14

Figure S1 | Manhattan and QQ plots for the iDXA GWAS. GWAS were conducted across the 15 iDXA traits, listed in Supplementary Data 1, excluding all non-white-British and related individuals, in a remaining cohort of approximately 392,535 individuals. Genome-wide Manhattan and QQ plots depict the observed SNP-trait associations and the distributions of the observed versus expected GWAS p-values, as follows; **a**, iDXA Android fat mass, **b**, iDXA Android fat mass index (FMI), **c**, iDXA Android fat-to-lean mass ratio (FMR), **d**, iDXA Android lean mass index (LMI), **e**, iDXA Apple fat, **f**, iDXA Gynoid fat mass, **g**, iDXA Leg FMI, **h**, iDXA Leg FMR, **i**, iDXA Pear fat, **j**, iDXA Segmental fat index (SFI), **k**, iDXA Subcutaneous fat, **l**, iDXA Total lean mass percentage, **m**, iDXA Trunk fat index, **n**, iDXA Trunk lean index, **o**, iDXA visceral adipose tissue FMI. GWAS summary statistics can be accessed via the University of Edinburgh's DataShare repository (https://doi.org/10.7488/ds/2973).

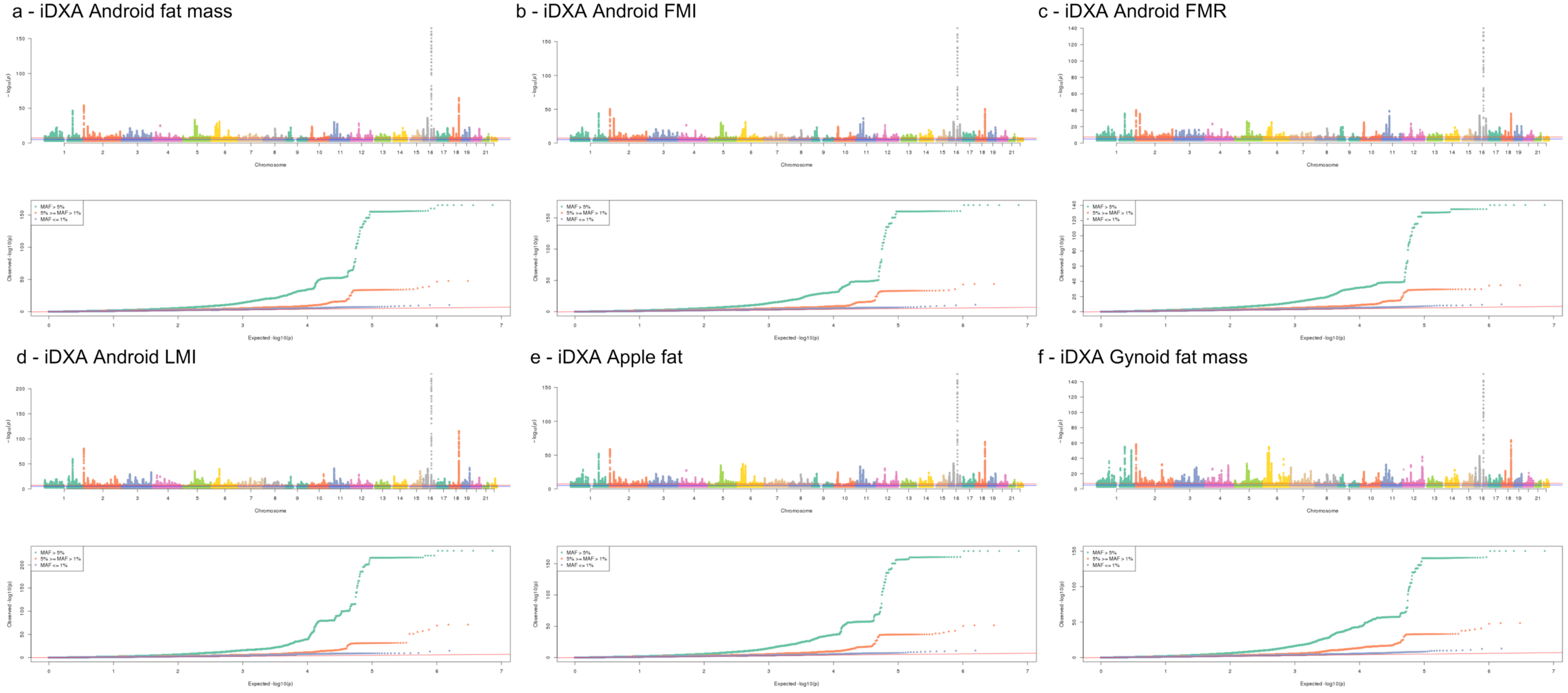

g - iDXA Leg FMI

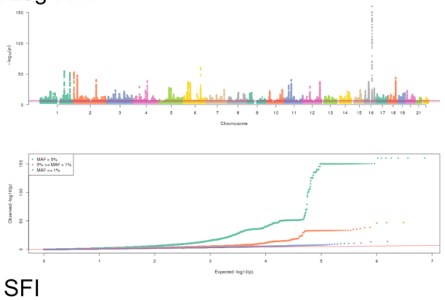

h - iDXA Leg FMR

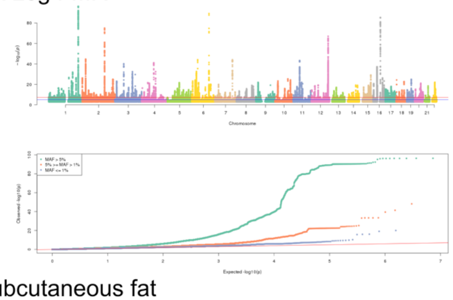

i - iDXA Pear fat

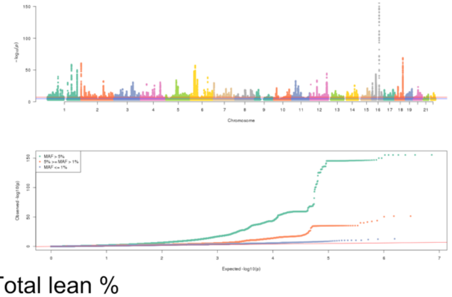

j - iDXA SFI

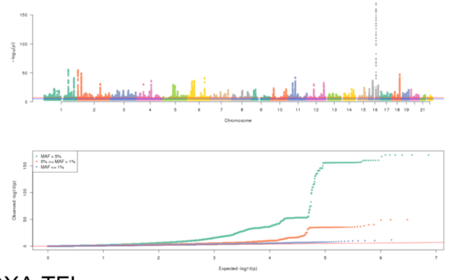

k - iDXA Subcutaneous fat

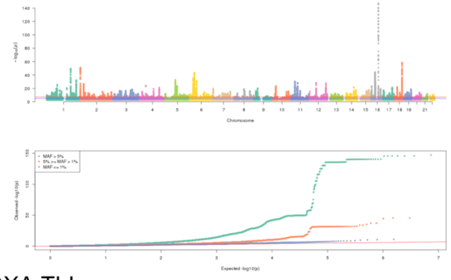

l - iDXA Total lean %

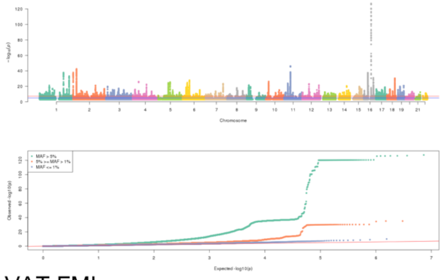

m - iDXA TFI

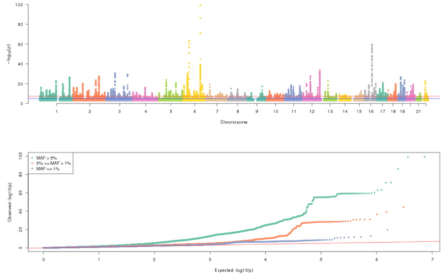

n - iDXA TLI

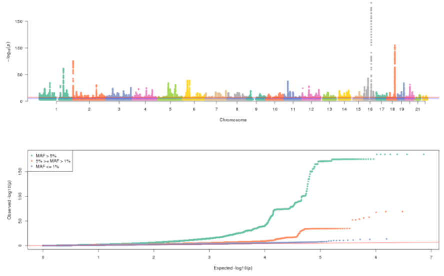

o - iDXA VAT FMI

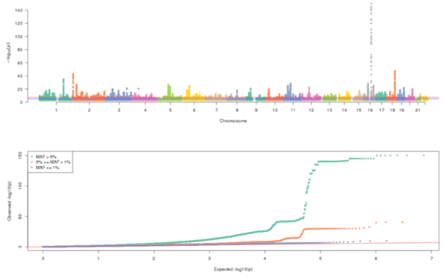

Figure S2 | iDXA GWAS tissue enrichment. Linkage-disequilibrium score regression to specifically expressed genes (LDSC-SEG) was used to test for enrichment of the iDXA GWAS data for genes specifically expressed throughout the GTEx tissues. The below test statistic represents the FDR-corrected P-values, as calculated by LDSC-SEG (which represent a one-sided test that the coefficient is greater than zero), corrected for the number of tested tissues (48) and multiplied by the effective number of tested traits (4). Extended output from this analysis can be found in Supplementary Data 2.

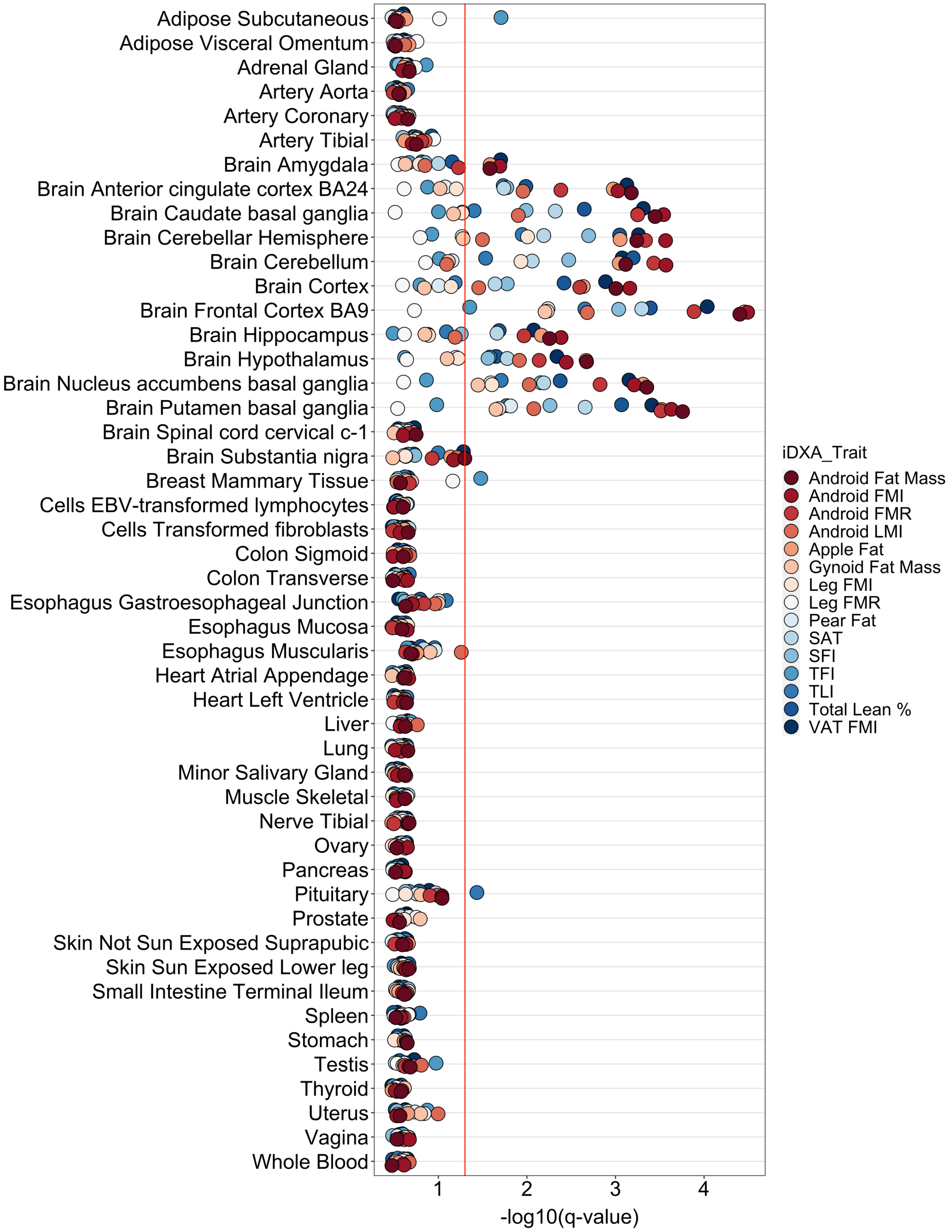

Figure S3 | Six out of 27 prioritised loci replicated in the DXA cohort MA at a 10% FDR. Locus zoom plots of GWAS statistics (two-sided) for the six replicated variants and surrounding loci (±500kb) as follows; **a**, locus containing PLA2G6/MAFF, **b**, locus containing CPS1, **c**, locus containing ACVR2B/EXOG, **d**, locus containing FBX036, **e**, locus containing ADAMTS14 and **f**, locus containing ACADVL/DLG4. Genomic locations and annotations given in GRCh37/hg19 and dbSNP build 150. GWAS summary statistics can be accessed via the University of Edinburgh's DataShare repository (https://doi.org/10.7488/ds/2973).

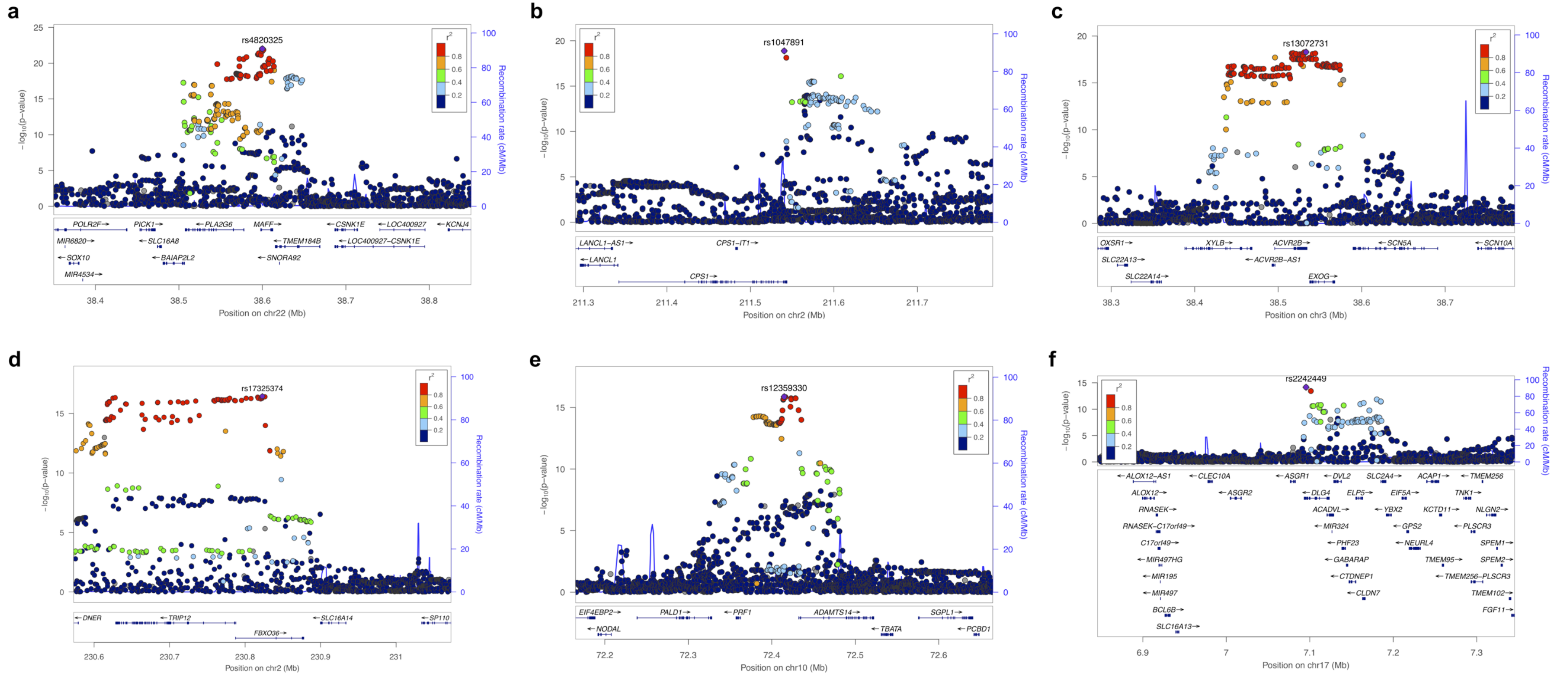

Figure S4 | Lookup of replicated signals in the BIA and anthropometric components of the iDXA imputation models. UKB summary statistics from Neale et al. accessed via Phenoscanner (see Methods). Symbols within each box indicate the direction of effect on the listed trait, aligned for the reported iDXA effect alleles as seen in Table 1. Extended output can be found in Supplementary Data 5.

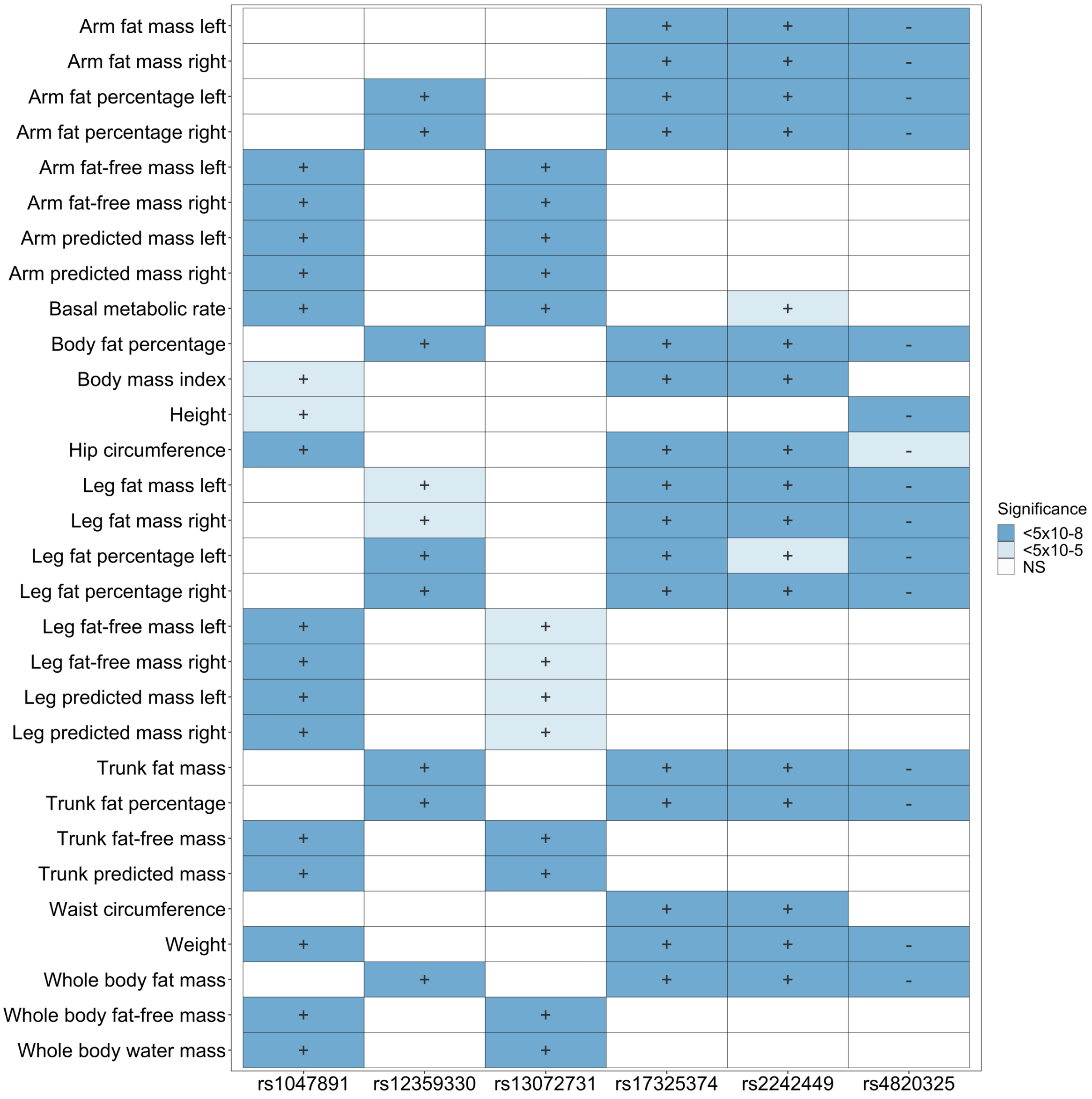

Figure S5 | Pleiotropic iDXA associations. **a**, GWAS statistics (two-sided) of each replicated signal across the 15 iDXA traits. Extended output can be found in Supplementary Data6. **b**, Summary of other pleiotropic iDXA associations among the 242 identified "novel" loci. Numbers on lower side of plot indicate how many other iDXA traits (other than the Discovery trait) each of the signals is genome-wide significant ($p<1.25x10^{-8}$) in. Extended output can be found in Supplementary Table 3.

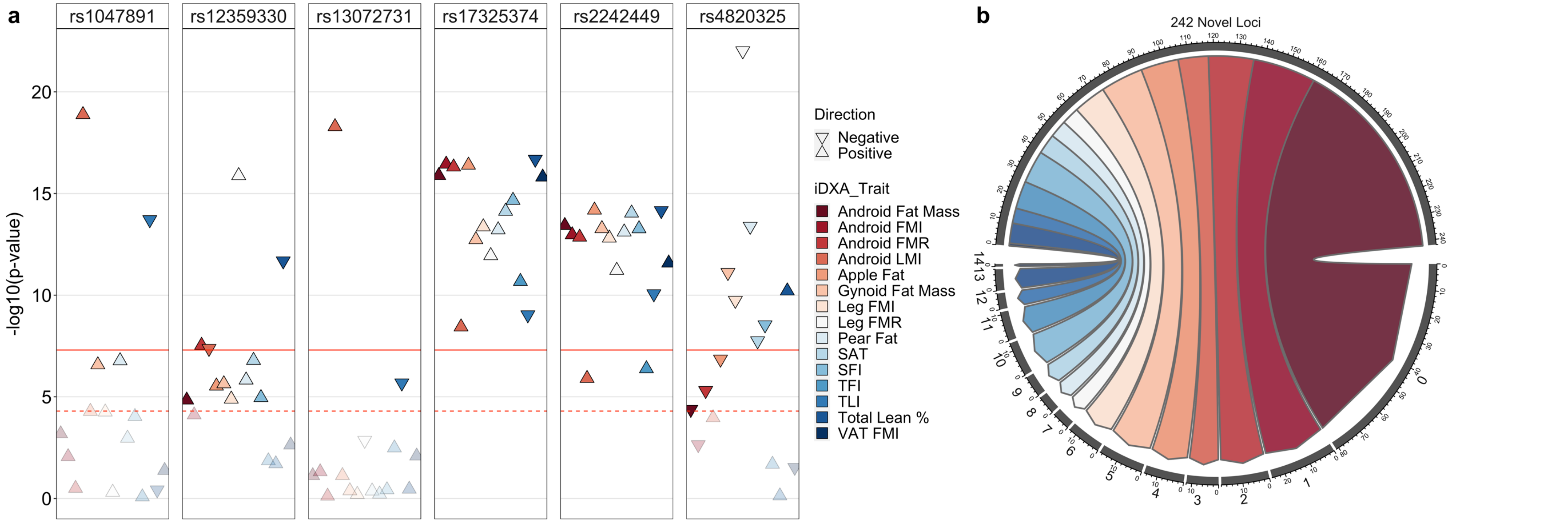

Figure S6 | Lookup of replicated signals in other ancestry groups. Comparison of the effects of each replicated signals on the Discovery iDXA GWAS trait across the other three broad ancestry groupings, shown as beta ± 95% confidence intervals. Sample sizes are Other white (n=29,015), Asian (n=10,920), Black (n=7,644) and Discovery (n=392,535). Extended output can be found in Supplementary Data 7.

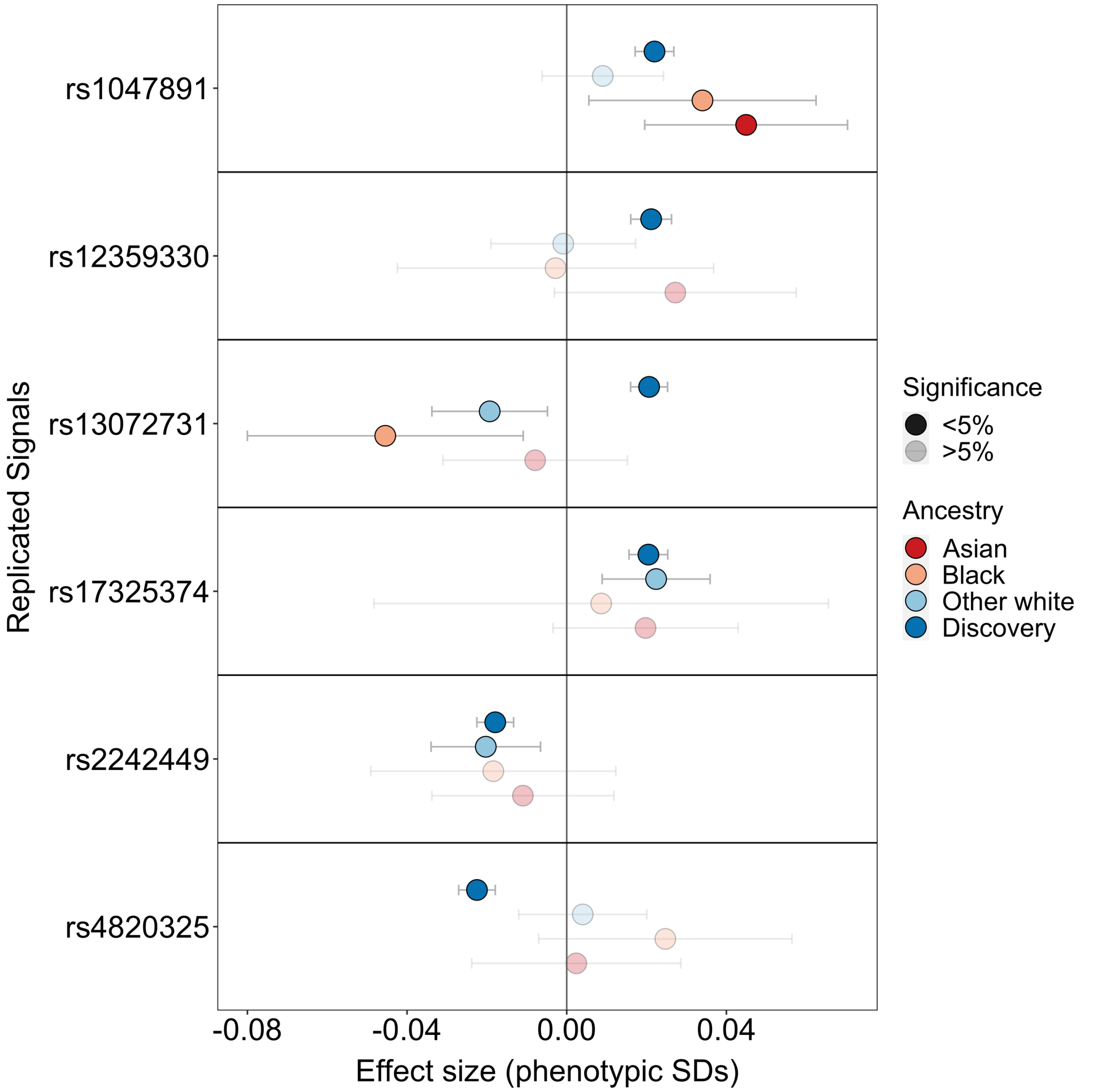

Figure S7 | Multiple causal gene candidates at most replicated loci. Each panel shows **a,** the SMR p-values (two-sided) for all GTEx (V7) tissues MA at which changes in gene expression colocalised with the association patterns and **b,** the direct eQTLs in the cross-tissue, at each of the 6 replicated loci. All shown have FDR<5% and P HEIDI>5%. Extended output can be found in Supplementary Data 9.

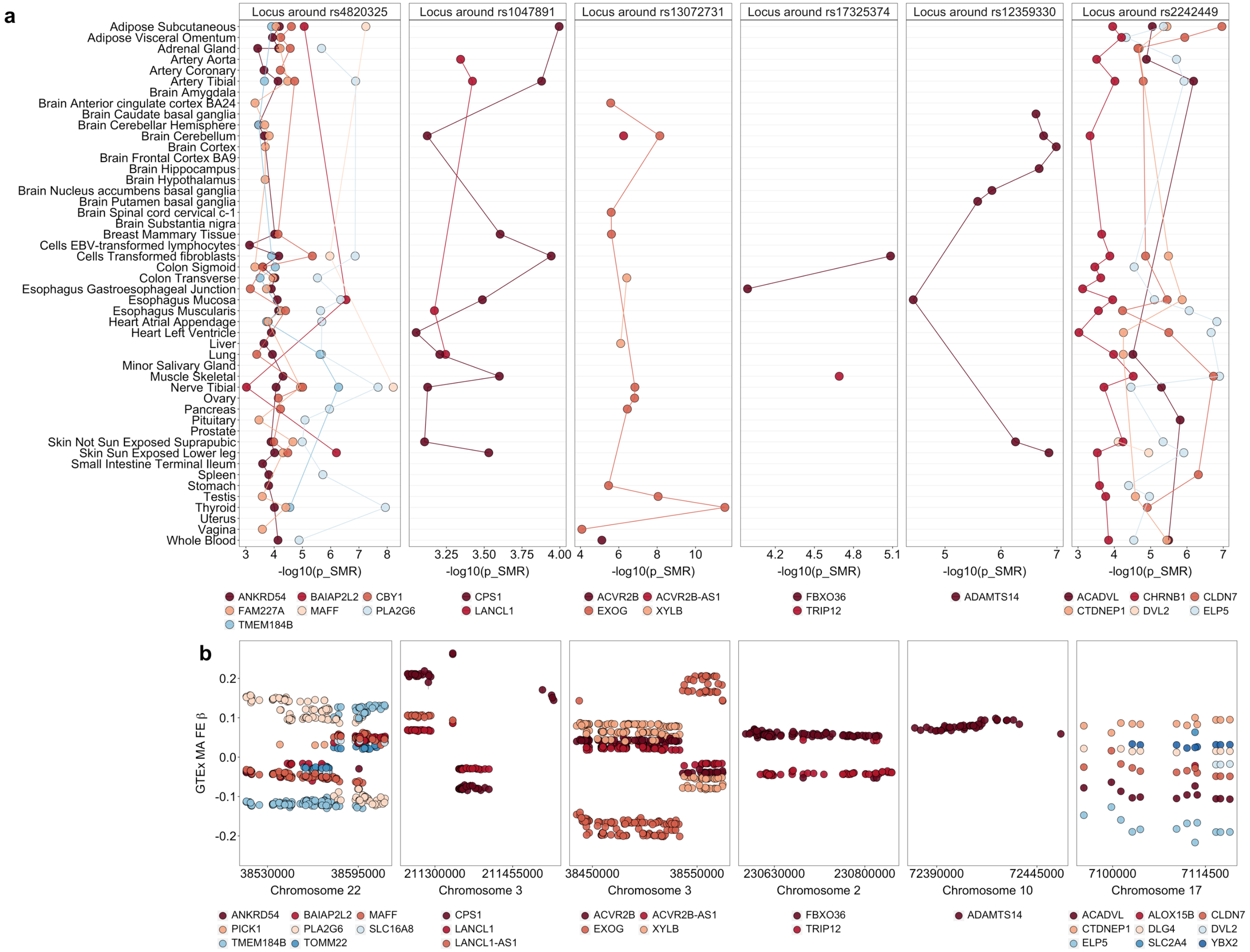

Figure S8 | Experimental design for the phenotyping of the *Adamts14*$^{+/-}$ and *Adamts14*$^{-/-}$ mice. Schematic representation of the experimental design for the in vivo metabolic characterisation of the *Adamts14*$^{+/-}$ (**a**) and *Adamts14*$^{-/-}$ (**b, c**) mice. All cohorts were exposed to HFD and weighed weekly for 13/6 weeks, with glucose homeostasis and body composition being assessed at the start and end of the HFD exposure, via oGTT and TD-NMR respectively. Heterozygous animals underwent both assessments at the midpoint of the experiment as well (6 weeks). Homozygous animals underwent indirect calorimetry (indicated by PM) at the start and end of the experiment. Tissues were collected at the end of the experiments for further analysis. Mouse outlines were downloaded via https://openclipart.org/detail/17558/simple-cartoon-mouse and modified for this work.

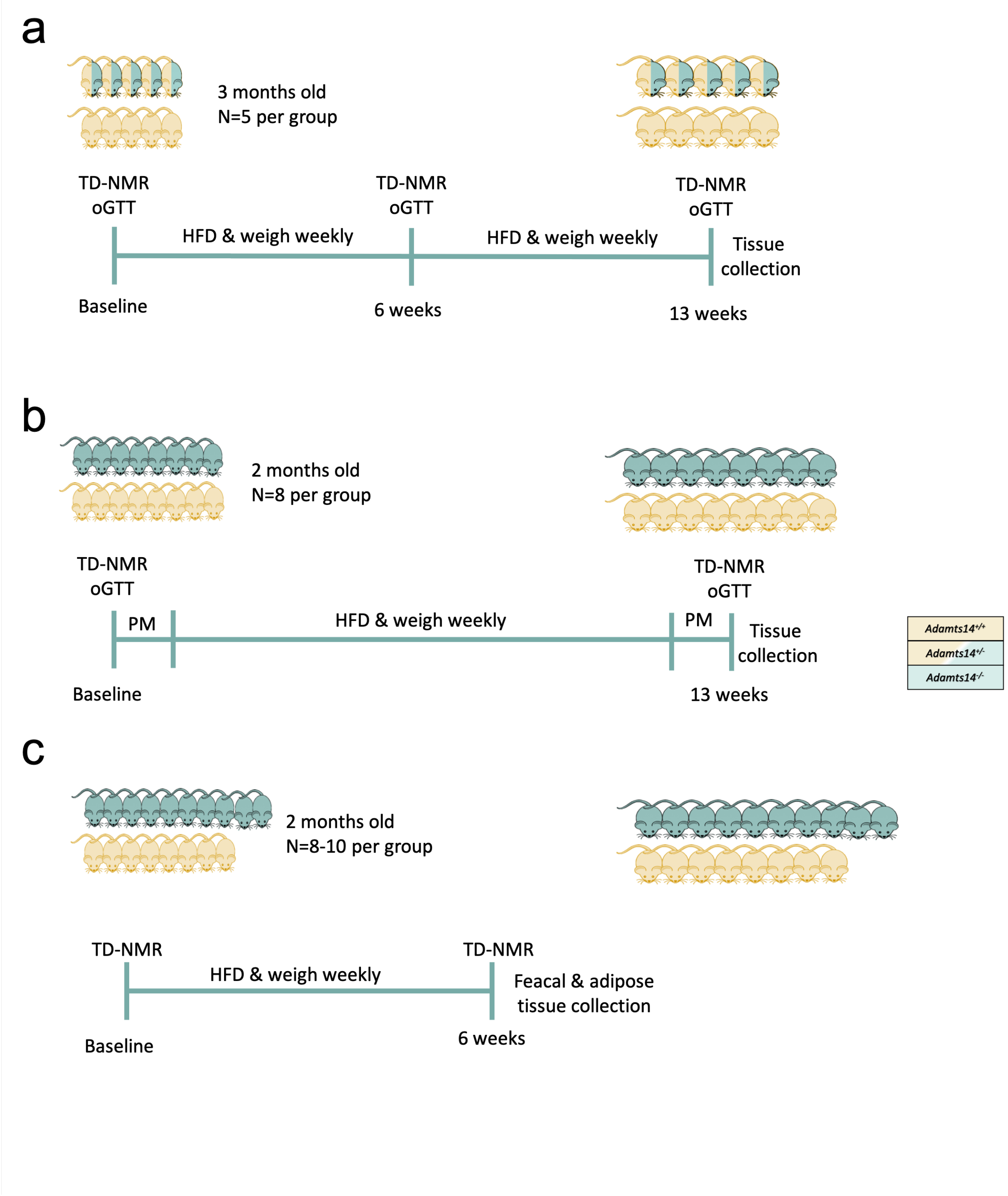

Figure S9 | The *Adamts14*$^{+/-}$ mouse was resistant to weight and fat gain and had improved glucose homeostasis under HFD conditions. a, Body weight of WT and heterozygous animals through 13 weeks of HFD, starting when the animals were 3 months old. **b**, *Post mortem* organ and tissue weights of the WT and *Adamts14*$^{+/-}$ mice. b-inset, Comparison of length of the left tibia, the mouse from nose to anus and the gut. **c**, Fat and lean mass changes before HFD and after 6 and 13 weeks. **d**, Oral glucose tolerance tests, circulating glucose levels and AUC comparison, at 0, 6 and 13 weeks of HFD. N=5 per genotype. Data expressed as mean ± se. T-tests done for direct comparisons in b, c and d and a linear mixed model with repeated measures was done over the whole experimental period of a, c and d. All tests were two-sided. Significance denoted as * for p<0.05, ** for p<0.01 and *** for p<0.001, while specific p-values were **a:** p = 0.09, **c:** p = 0.033, **d:** p = 0.012 and p = 0.03 respectively. Extended output can be found in Supplementary Data 10.

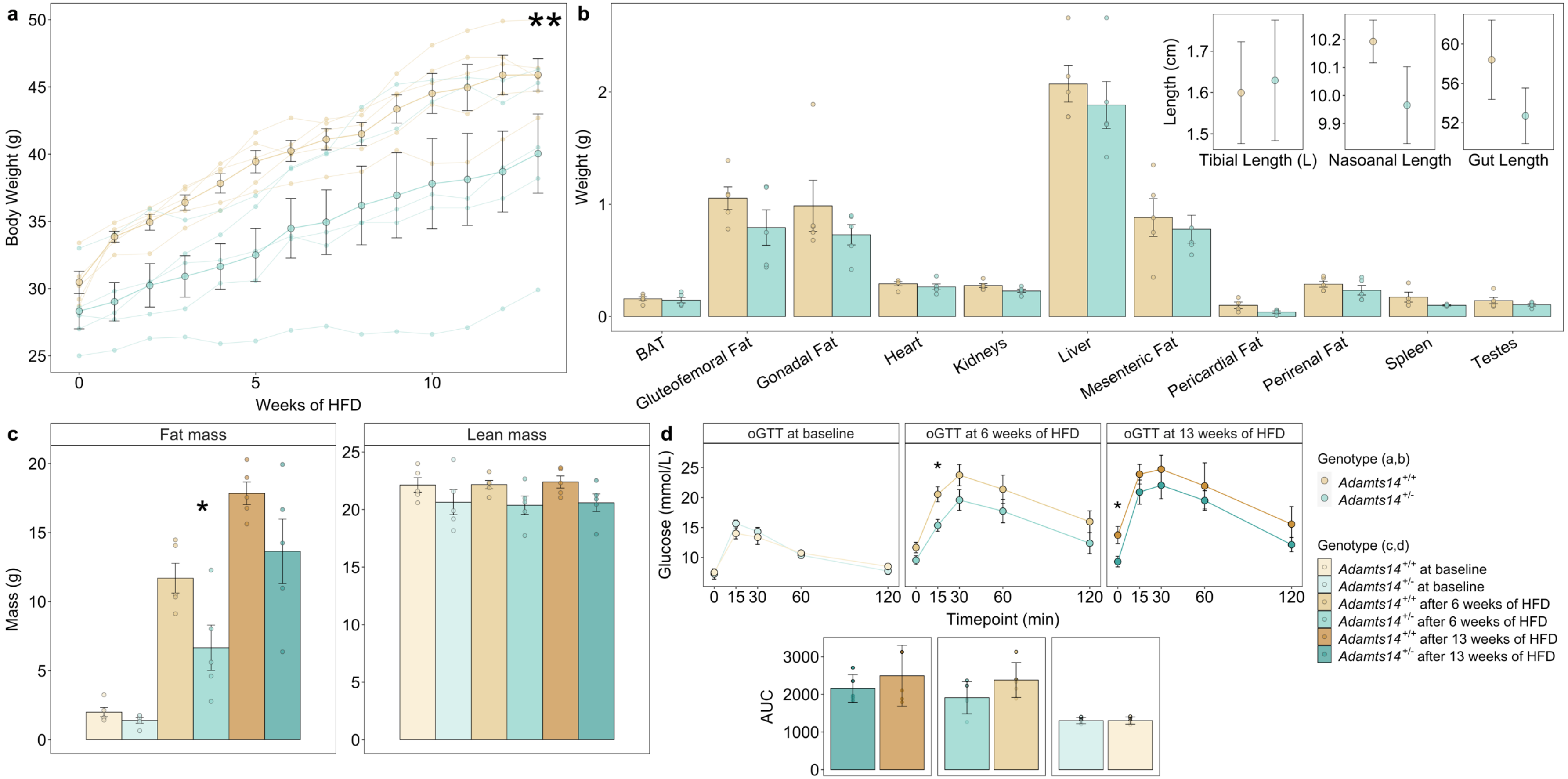

Figure S10 | The *Adamts14*$^{-/-}$ mouse shows no differences in adiposity and glucose homeostasis. a, *Post mortem* organ and tissue weights of the WT and *Adamts14*$^{-/-}$ mice. **a**-inset, Comparison of length of the left tibia, the mouse from to anus and the gut (two-sided t-test, tibial length: p = 0.008; gut length: p = 0.009). **b**, Oral glucose tolerance tests, at before and after the HFD exposure. **c**, corresponding AUC data for the two oGTTs. **d**, Fat and lean mass changes before and after 13 weeks of HFD. N=8 per genotype initially, down to 6 WT and 7 *Adamts14*$^{-/-}$ by 13 weeks. Data expressed as mean ± se. T-tests done for direct comparisons in b, c and d and a linear mixed model done over the whole experimental period of a and c. Significance denoted as * for p<0.05, ** for p<0.01 and *** for p<0.001. Extended output can be found in Supplementary Data 11.

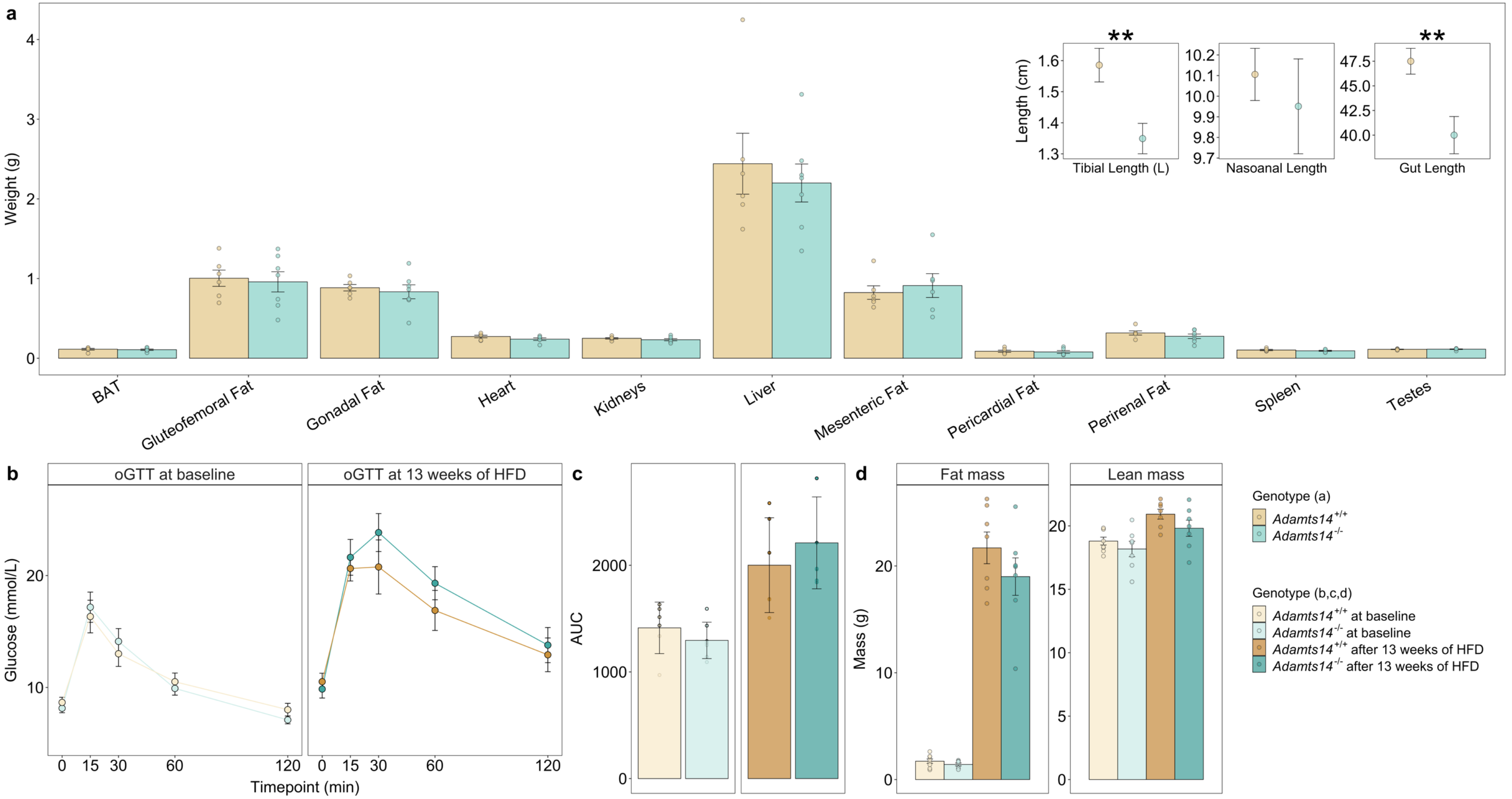

Figure S11 | No histomorphological difference seen between *Adamts14*$^{-/-}$ and *Adamts14*$^{+/+}$ mice. a, Sections of gluteofemoral fat, gonadal fat, brown adipose tissue (BAT), liver and back skin, accordingly, stained with picrosirius red (PSR). Scale bar given in skin. **b**, Collagen content quantification in the gluteofemoral and gonadal fat depots of WT and *Adamts14*$^{-/-}$ animals after 13 and 6 weeks of HFD exposure. Data expressed as mean ± se. N=6 WT (31 gluteofemoral and 28 gonadal images) and 7 *Adamts14*$^{-/-}$ (34 gluteofemoral and 34 gonadal images) for the 13 weeks cohort and 8 WT (47 gluteofemoral and 44 gonadal images) and 10 *Adamts14*$^{-/-}$ (51 gluteofemoral and 47 gonadal images) for the 6 weeks cohort. At least 3 independent images were quantified per depot per animal. Extended output can be found in Supplementary Data 15.

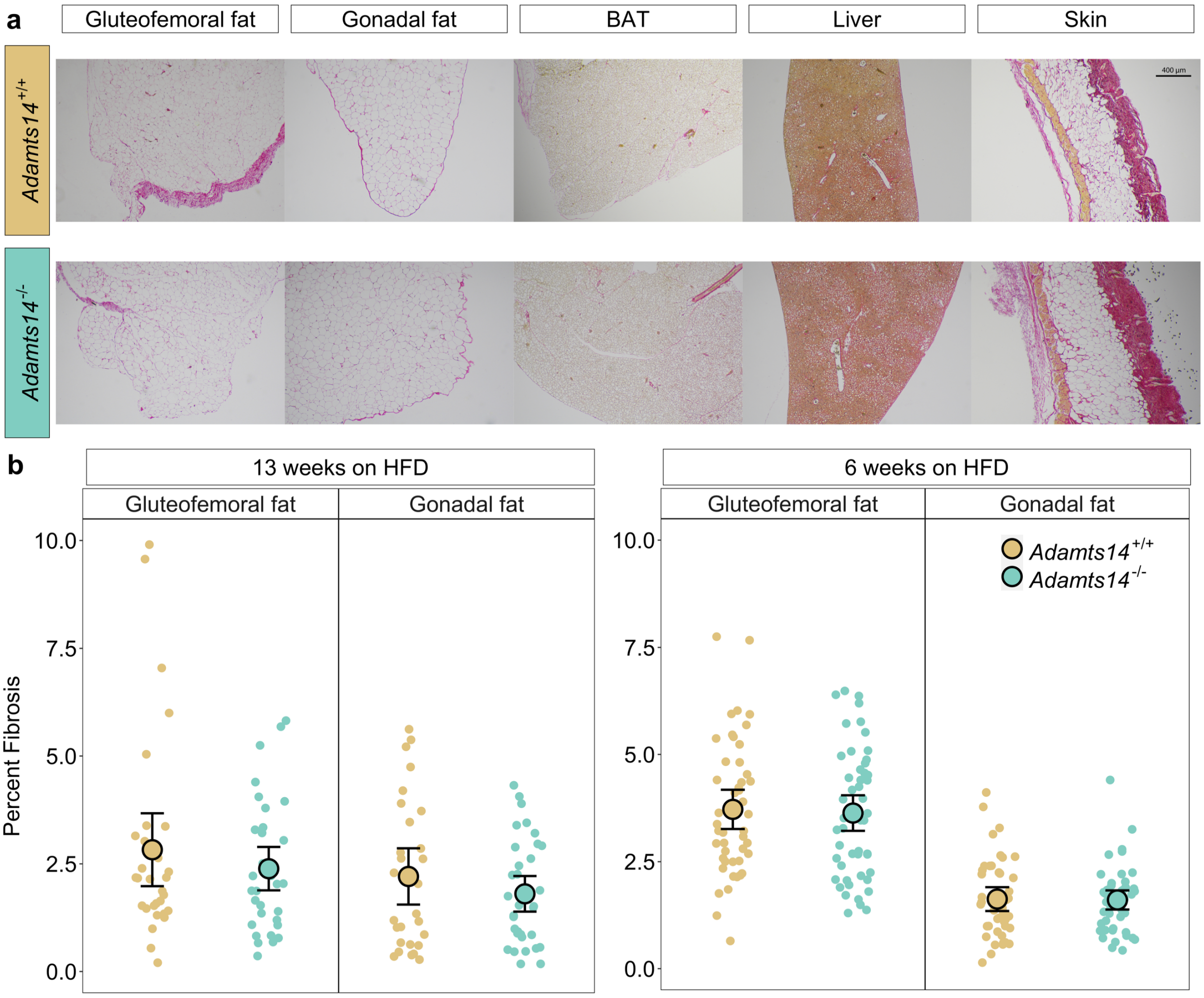

Figure S12 | No apparent differences in nutrient absorption after 6 weeks of HFD exposure. a, Body weight of WT and *Adamts14*$^{-/-}$ animals through 6 weeks of HFD. Data expressed as mean ± se. **b**, Fat and lean mass changes before and after the HFD exposure. Data expressed as mean ± se. **c**, Fecal energy content of the WT and *Adamts14*$^{-/-}$ animals after the 6 week exposure to the HFD. Median, 25th and 75th centile as indicated by box centre, minima, and maxima, accordingly, with whiskers encompassing the full spread of the data. N=8 WT and 10 *Adamts14*$^{-/-}$. T-tests done for direct comparisons. Extended output can be found in Supplementary Data 14.

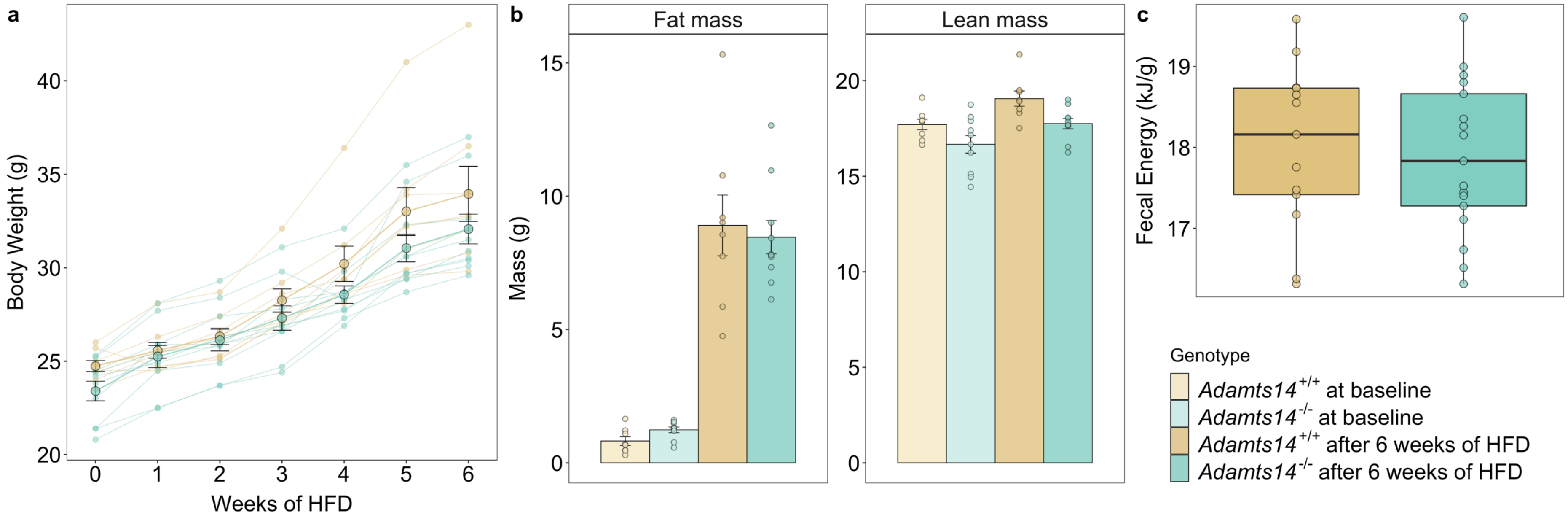

Supplement: Supplementary file 1 — Supplementary Information [file 41467_2022_35563_MOESM1_ESM.pdf]
