# Supplementary material for: Large scale phenotype imputation and in vivo functional validation implicate ADAMTS14 as an adiposity gene
Source: Nat Commun. 2023 Jan 19;14:307. doi: 10.1038/s41467-022-35563-0 (PMC9852585; doi:10.1038/s41467-022-35563-0)
Supplement: Supplementary file 2 — Description of Additional Supplementary Files [file 41467_2022_35563_MOESM2_ESM.pdf]

Description of Additional Supplementary Files

File Name: Supplementary Data 1

Description: Correlations between measured DXA and iDXA phenotypes. Pearson correlation coefficients (R) calculated for the 4,400 participants with both DXA and iDXA, separately for the two sexes, ranged from 51 to 91%, but were 81% on average. Height measures used in cm and mass in grams. Abbreviations refer to: FMI; fat mass index, FMR; fat-to-lean mass ratio, LMI; lean mass index.

File Name: Supplementary Data 2

Description: iDXA GWAS tissue enrichment. Linkage-disequilibrium score regression to specifically expressed genes (LDSC-SEG) was used to test for enrichment of the iDXA GWAS data for genes specifically expressed throughout the GTEx tissues. The test statistics, as calculated by LDSC-SEG represent a one-sided test that the coefficient is greater than zero. P-values were FDR-corrected (q-val) for the number of tested tissues (48) and multiplied by the effective number of tested traits (4).

File Name: Supplementary Data 3

Description: Prioritised loci and pleiotropic iDXA associations SNP summary statistics for the 242 prioritised "novel" SNPs in their respective iDXA Discovery trait. Genomic locations and annotations given in GRCh37/hg19 and dbSNP build 150. chr_pos denotes the chromosome and chromosomal location of each variant. Other headers as follows; a1; effect allele, a0; other allele, freq1; frequency of a1, beta1; effect per copy of a1, se; standard error of beta1 and p; p-value (two-sided) for the association, N Other Assocs; number of other iDXA GWAS that this SNP is genome-wide significant in, Other iDXA Traits; list of said traits.

File Name: Supplementary Data 4

Description: DXA cohort replication summary statistics SNP summary statistics for the 27 tested SNPs in the 4 replication cohorts. Column contents as specified in SD3.

File Name: Supplementary Data 5

Description: Lookup of replicated signals in the BIA and anthropometric components of the iDXA imputation models. Phenoscanner output displaying the association statistics (two-sided) observed between each of the six replicated and the anthropometric and BIA components of the imputation models, via Neale et al. UKB summary statistics.

File Name: Supplementary Data 6

Description: Lookup of replicated signals across the 15 iDXA GWAS. Association statistics observed between each of the six replicated and the 15 iDXA traits. Column contents as specified in SD4.

File Name: Supplementary Data 7

Description: Lookup of replicated signals in other ancestry group Association statistics (two-sided) observed between each of the six replicated and the corresponding Discovery iDXA trait in participants from the further 3 broad ancestry groups. Column contents as specified in SD3.

File Name: Supplementary Data 8

Description: Colocalisation of iDXA GWAS and eQTL data at the ADAMTS14 locus. Colocalisation was confirmed in all tissues where HEIDI-test p-value>5% and/or PP H4 ABF is >0.75. Headers as follows, GTEx_tissue; denotes which GTEx tissues the following correspond to, Gene; denotes the gene symbol, h0.pp; posterior probability that neither trait has a genetic association in the region, h1.pp; posterior probability that only the iDXA GWAS has a genetic association in the region, h2.pp; posterior probability that only the eQTL has a genetic association in the region, h3.pp; posterior probability that both traits are associated, but with different causal variants, h4.pp; posterior probability that both traits are associated and share a single causal variant, p_SMR; two-sided p-value for the SMR test, p_HEIDI; p-value for the HEIDI test, nsnp_HEIDI; number of SNPs included in the HEIDI test.

File Name: Supplementary Data 9

Description: eQTL analyses across the six replicated iDXA signals. Extended output from eQTL analyses, across the 6 replicated loci. Headers as follows; Locus around; denotes the replicated iDXA GWAS signals, gtex_tissue; denotes GTEx tissue to which the following correspond to, probeID; denotes the ID of the probe, ProbeChr; denotes denotes the chromosome where probeID lies, Gene; denotes the gene symbol of the gene where the probe binds, Probe_bp; denotes the chromosomal position of the probe, topSNP; denotes the top chosen SMR SNP, topSNP_chr; denotes the chromosome where topSNP lies, topSNP_bp; denotes the chromosomal position of topSNP in GRCh37, A1; denotes the effect allele of topSNP, A2; denotes the other allele of topSNP, Freq; denotes the frequency of A1, b_GWAS; denotes effect size estimate of topSNP in the GWAS data, se_GWAS; denotes standard error of the b_GWAS, p_GWAS; denotes denotes p-value of topSNP in the GWAS data, b_eQTL; denotes effect size estimate of topSNP in the eQTL data, se_eQTL; denotes standard error of the b_eQTL, p_eQTL; denotes p-value of topSNP in the eQTL data, b_SMR; denotes effect

size estimate of the SMR test, se_SMR; denotes standard error of the b_SMR, p_SMR; denotes p-value of the SMR test, p_HEIDI; denotes p-value of the HEIDI test, nsnp_HEIDI; denotes number of SNPs included in the HEIDI test.

File Name: Supplementary Data 10

Description: Metabolic phenotyping data from the WT-heterozygous 13-week cohort of mice. Metabolic phenotyping data from the WT-heterozygous 13-week cohort of mice. Headers as follows, id; signifies the id of the animal, genotype; wt/het/ko, signifies the genotype of the animal, w[0-9]; signifies the weight of the anumal during the corresponding week of HFD exposure, in grams, tdnmr[0-9]_[tissue_type]; signifies the corresponding tissue type mass during the corresponding experimental timepoint, in grams, gtt[0-9]_t[0-9]; signifies the circulating glucose levels during the corresponding timepoint and experiment, in mmol/L, BAT; Brown Adipose Tissue weight at time of post mortem, in grams, equivalently for other measured tissues and in cm where length is specified, litter_coded; litter code for id.

File Name: Supplementary Data 11

Description: Metabolic phenotyping data from the WT-homozygous 13-week cohort of mice. Headers as in SD10.

File Name: Supplementary Data 12

Description: 13 week homozygous mouse cohort indirect calorimetry. Headers as follows, id; signifies the id of the animal, genotype; wt/ko, signifies the genotype of the animal, time; time of day when measurement was taken - starts at 709 am and finishes at 654am the following day, consistent with the light and dark phases on the facility where the animals are being kept, the following four measurements are available pre- (chow_*) and post-HFD treatment (hfd_*), i.e. at baseline and after 12 weeks of exposure to the diet; RER; denotes respiratory exchange ratio, H(3); denotes uncorrected energy expenditure, in watts, XT+YT; denotes detected activity as laser beam breaks, Feed; denotes amount of food consumed, in grams.

File Name: Supplementary Data 13

Description: 13 week homozygous mouse cohort adipocyte size quantification. Headers as follows, label; denotes the image pertaining to the following measure, id; signifies the id of the animal, genotype; wt/ko, signifies the genotype of the animal, tissue; denotes the type of tissue - gon for gonadal and glut for gluteofemoral, area; denotes the area covered by each individual adipocyte within this image and count; denotes the total number of adipocytes captured within each image.

File Name: Supplementary Data 14

Description: Metabolic phenotyping data from the WT-homozygous 6-week cohort of mice. Headers as in SD10 and faecal_wet_weight; denoting the weight of the faecal tissue prior to drying, faecal_dry_weight; weight after drying, faecal_notes; denote whether amount of dry sample enough for two replicates, faecal_Energy_1; denotes energy produced from first replicate; faecal_Energy_2; denotes energy produced from second replicate if sufficient material was collected.

File Name: Supplementary Data 15

Description: 6 and 13 week homozygous mouse cohort collagen content. Quantification of collagen content on PSR-stained adipose sections from cohorts in SD12 and SD14. Headers as follows; cohort; denotes the experimental cohort, i.e. 13 or 6 weeks, image; denotes the image pertaining to the following measure, id; signifies the id of the animal, genotype; wt/ko, signifies the genotype of the animal, tissue; denotes the type of tissue - gon for gonadal and glut for gluteofemoral, psr_*_pc and psr_*_area; denote the percentage of image or area of the image (in pixels) that was either PSR positive, i.e. stained with PSR indicating the presence of fibrillar collagen I or III, PSR negative, or other. psr_pos_pc was used for genotype comparisons.

File Name: Supplementary Data 16

Description: Known "obesity" associations from the GWAS catalog used for locus filtering. All SNP associations (two-sided) published on the GWAS catalog, falling under the "obesity" trait umbrella, as seen at the time of original locus prioritisation (circa April 2018). Genomic positions are given in GRCh37.

File Name: Supplementary Data 17

Description: Updated "obesity" associations from the GWAS catalog. All SNP associations (two-sided) published on the GWAS catalog, falling under the "obesity" trait umbrella, as seen in July 2022. Annotations to the six replicated loci within this work, done purely on the basis of signal location, within ±500kb of the 6 prioritised loci. Genomic positions are given in GRCh38.
