# Supplementary material for: Large scale phenotype imputation and in vivo functional validation implicate ADAMTS14 as an adiposity gene
Source: Nat Commun. 2023 Jan 19;14:307. doi: 10.1038/s41467-022-35563-0 (PMC9852585; doi:10.1038/s41467-022-35563-0)
Supplement: Supplementary file 4 — Reporting Summary [file 41467_2022_35563_MOESM4_ESM.pdf]

nature portfolio

Corresponding author(s): Katherine A Kentistou & Nicholas M Morton

Last updated by author(s): 2022/11/11

Reporting Summary

Nature Portfolio wishes to improve the reproducibility of the work that we publish. This form provides structure for consistency and transparency in reporting. For further information on Nature Portfolio policies, see our Editorial Policies and the Editorial Policy Checklist.

Statistics

For all statistical analyses, confirm that the following items are present in the figure legend, table legend, main text, or Methods section.

n/a	Confirmed	
☐	☒	The exact sample size (n) for each experimental group/condition, given as a discrete number and unit of measurement
☐	☒	A statement on whether measurements were taken from distinct samples or whether the same sample was measured repeatedly
☐	☒	The statistical test(s) used AND whether they are one- or two-sided<br>*Only common tests should be described solely by name; describe more complex techniques in the Methods section.*
☐	☒	A description of all covariates tested
☐	☒	A description of any assumptions or corrections, such as tests of normality and adjustment for multiple comparisons
☐	☒	A full description of the statistical parameters including central tendency (e.g. means) or other basic estimates (e.g. regression coefficient) AND variation (e.g. standard deviation) or associated estimates of uncertainty (e.g. confidence intervals)
☐	☒	For null hypothesis testing, the test statistic (e.g. F, t, r) with confidence intervals, effect sizes, degrees of freedom and P value noted<br>*Give P values as exact values whenever suitable.*
☒	☐	For Bayesian analysis, information on the choice of priors and Markov chain Monte Carlo settings
☐	☒	For hierarchical and complex designs, identification of the appropriate level for tests and full reporting of outcomes
☐	☒	Estimates of effect sizes (e.g. Cohen's d, Pearson's r), indicating how they were calculated

Our web collection on statistics for biologists contains articles on many of the points above.

Software and code

Policy information about availability of computer code

Data collection	All human cohort data had been previously collected.
Data analysis	Adiposoft plugin within ImageJ (1.53c), RTrees within QuPath (0.3.2).<br>R (v4.0.1); including PhenoSpD (v1.0.0), coloc (v3.2.1), glmmTMB (v1.0.2.1),REGSCAN (v0.5), SMR-HEIDI (v0.68), GTEx (v7), LDSC-SEG (1.0.1).

For manuscripts utilizing custom algorithms or software that are central to the research but not yet described in published literature, software must be made available to editors and reviewers. We strongly encourage code deposition in a community repository (e.g. GitHub). See the Nature Portfolio guidelines for submitting code & software for further information.

Data

Policy information about availability of data

All manuscripts must include a data availability statement. This statement should provide the following information, where applicable:

- Accession codes, unique identifiers, or web links for publicly available datasets
- A description of any restrictions on data availability
- For clinical datasets or third party data, please ensure that the statement adheres to our policy

This research has been conducted using the UK Biobank Resource, approved under application 19655. Processed cross-tissue and tissue-wide GTEx data v7 are available on the GTEx portal: https://gtexportal.org. GWAS summary statistics are available from the University of Edinburgh's DataShare repository (https://doi.org/10.7488/ds/2973). All other data supporting the findings of this study is available through the supplement.

Human research participants

Policy information about studies involving human research participants and Sex and Gender in Research.

Reporting on sex and gender	Data was analysed and/or used separetely on the basis of sex only when there was a biological reason to do so, i.e. when accounting for different body composition etc. Any such instances are explicitly described in the methods text.<br>The term gender is used when describing instances where participants genetic sex and self-described gender differed.
Population characteristics	The UKB is a large population-based cohort with over 500,000 participants, aged 40 to 69, from across the UK. Its aim is to improve the prevention, diagnosis and treatment of serious and life-threatening illnesses affecting people of middle- and old-age. UKB includes extensive phenotypic and genotypic data on its participants, including questionnaire data, physical measures, blood and urine sample assays, accelerometry, multimodal imaging, genome-wide genotyping and longitudinal follow-up.<br>The Orkney Complex Disease Study – ORCADES cohort is a population-based isolate that includes 2215 individuals. Its aim is to characterise the genetic and epidemiological components that underlie quantitative traits and diseases in the Orkney Islands of Scotland. The ORCADES cohort includes 2,215 participants aged 18 and over, 1,256 of which underwent full body DXA composition analysis.<br>The European Prospective Investigation of Cancer (EPIC) is a large multi-centre prospective cohort study that focuses on the connection between diet, lifestyle factors and cancer. The Norfolk cohort includes 25,639 individuals, aged 40 to 79, living in Norwich and the surrounding towns and rural areas.<br>The Fenland Study is an ongoing population-based cohort study, that includes 12,435 adults aged 29–65 years in Cambridgeshire, UK.
Recruitment	Participants were invited to join UK Biobank over a period of 5 years, from 2006 to 2010.<br>For ORCADES, individuals of all ages were recruited based on the basis of their Orcadian heritage and have at least two Orcadian grandparents, thus maintaining the homogeneous genetic background of the cohort. Data collection was carried out between 2005 and 2011 in Orkney by trained research nurses.<br>EPIC-Norfolk participants were recruited between 1993 and 1997 and have been contributing information about their lifestyle and health through questionnaires and health checks for over two decades.<br>Volunteers for the Fenland study were recruited from general practice registers between 2005 and 2015.
Ethics oversight	All UK Biobank participants gave written informed consent and the study was approved by the North West Multicentre Research Ethics Committee.<br>The Orkney Research Ethics Committee and North of Scotland Local Research Ethics Committee granted the study ethical approval and all participants individuals gave written, informed consent prior to participating in any research, such as broad-ranging health and disease or population research, including biobanking of samples or record linkage to hospital admissions or to other records.<br>All EPIC-Norfolk participants gave signed informed consent and The Norwich District Health Authority Ethics Committee approved the study.<br>The Fenland study was approved by the Cambridge Local Research Ethics Committee and all participants gave written informed consent.

Note that full information on the approval of the study protocol must also be provided in the manuscript.

Field-specific reporting

Please select the one below that is the best fit for your research. If you are not sure, read the appropriate sections before making your selection.

☒ Life sciences ☐ Behavioural & social sciences ☐ Ecological, evolutionary & environmental sciences

For a reference copy of the document with all sections, see nature.com/documents/nr-reporting-summary-flat.pdf

Life sciences study design

All studies must disclose on these points even when the disclosure is negative.

Sample size	Information about human cohort sample sizes is available in the Methods and Supplement. The UK Biobank is a publicly available population-based cohort and, as such, the sample size was chosen based on the sample size in the dataset. In replication analyses, loci were prioritised for replication based on the maximum replication cohort size that could be attained via collaborators, under the assumption that the effect sizes observed in discovery and replication would be equal.<br>Information about animal cohort sample sizes is available in Supplementary Tables 10-15. Sample sizes could not be determined a priori, as the anticipated effect of the gene knockout on the queried phenotypes was unknown.
Data exclusions	UK Biobank data was filtered at the phenotype and genotype level, as seen in the relevant Methods sections. Briefly, participants were genotyped under two Affymetrix arrays, which show a 96% SNP overlap and resulted in 820,967 genetic markers being genotyped. SNPs were excluded on the basis of missingness and departure from Hardy-Weinberg equilibrium. SNPs were also excluded if they had a minor allele frequency smaller than 0.001 and an imputation quality score smaller than 0.4. Prior to GWAS, related and non-white-British individuals and all individuals with DXA phenotypes measured were excluded. The latter were excluded so that they can later be used to form a separate subsection of the DXA replication cohort. Following that, sex-separated log-transformed phenotypic values lying further than six standard

deviations on either side of the population mean were removed. After correcting all GWAS phenotypes for age, assessment centre, geographical coordinates, Townsend deprivation index, educational attainment, the first 20 principal components, genotyping array and batch, the resulting residuals were inverse rank transformed and values lying four standard deviations at either side of the population mean were removed.
No animal data was excluded.

Replication	Replication of SNPs associating with predicted DXA phenotypes was sought in separate cohorts with measured DXA phenotypes, in a total replication cohort size of 17,787 DXA participants. Replication was considered successful when the FDR-corrected one-sided p-value was <0.1 in replication and the direction of effect was consistent with the one observed in discovery.
Randomization	Associations were controlled by adjusting for potentially confounding covariates, such as ancestry principal components, geographic location, Townsend deprivation index, etc, as seen above and in the relevant Methods sections.
Blinding	All data was anonymised.

Reporting for specific materials, systems and methods

We require information from authors about some types of materials, experimental systems and methods used in many studies. Here, indicate whether each material, system or method listed is relevant to your study. If you are not sure if a list item applies to your research, read the appropriate section before selecting a response.

Materials & experimental systems

n/a	Involved in the study	
☒	☐	Antibodies
☒	☐	Eukaryotic cell lines
☒	☐	Palaeontology and archaeology
☐	☒	Animals and other organisms
☒	☐	Clinical data
☒	☐	Dual use research of concern

Methods

n/a	Involved in the study	
☒	☐	ChIP-seq
☒	☐	Flow cytometry
☒	☐	MRI-based neuroimaging

Animals and other research organisms

Policy information about studies involving animals; ARRIVE guidelines recommended for reporting animal research, and Sex and Gender in Research

Laboratory animals	Male C57BL/6J mice at 2 or 3 months of age were used for all experiments and were maintained single-housed in either standard or individually ventilated cages with ad libitum access to food and water at the Little France BRR facility.
Wild animals	No wild animals were used.
Reporting on sex	Male mice were used for experiments, as the human genetic findings did not indicate and sex-heterogeneity.
Field-collected samples	No field-collected samples were used.
Ethics oversight	All animal studies in this manuscript were licensed by the UK Home Office under project license PPL 60/8117, appropriate PILs granted under the Home Office Scientific Procedures (Animals) Act 1983 and after full ethical review by the University of Edinburgh Biological Sciences Services.

Note that full information on the approval of the study protocol must also be provided in the manuscript.
